# Differential diagnosis of pulmonary sarcoidosis: a review

**DOI:** 10.3389/fmed.2023.1150751

**Published:** 2023-05-12

**Authors:** Dominique Valeyre, Michel Brauner, Jean-François Bernaudin, Etienne Carbonnelle, Boris Duchemann, Cécile Rotenberg, Ingrid Berger, Antoine Martin, Hilario Nunes, Jean-Marc Naccache, Florence Jeny

**Affiliations:** ^1^Pulmonology Department, Groupe Hospitalier Paris Saint Joseph, Paris, France; ^2^INSERM UMR 1272, Sorbonne University Paris-Nord, Paris, France; ^3^Radiology Department, Avicenne University Hospital, Bobigny, France; ^4^Faculté de Médecine, Sorbonne University Paris, Paris, France; ^5^Microbiology Department, Avicenne University Hospital, Bobigny, France; ^6^Thoracic and Oncology Department, Avicenne University Hospital, Bobigny, France; ^7^Pulmonology Department, Avicenne University Hospital, Bobigny, France; ^8^Pathology Department, Avicenne University Hospital, Bobigny, France

**Keywords:** sarcoidosis, differential diagnosis, granuloma, tuberculosis, thoracic computed tomography, pathology, microbiology

## Abstract

Diagnosing pulmonary sarcoidosis raises challenges due to both the absence of a specific diagnostic criterion and the varied presentations capable of mimicking many other conditions. The aim of this review is to help non-sarcoidosis experts establish optimal differential-diagnosis strategies tailored to each situation. Alternative granulomatous diseases that must be ruled out include infections (notably tuberculosis, nontuberculous mycobacterial infections, and histoplasmosis), chronic beryllium disease, hypersensitivity pneumonitis, granulomatous talcosis, drug-induced granulomatosis (notably due to TNF-a antagonists, immune checkpoint inhibitors, targeted therapies, and interferons), immune deficiencies, genetic disorders (Blau syndrome), Crohn’s disease, granulomatosis with polyangiitis, eosinophilic granulomatosis with polyangiitis, and malignancy-associated granulomatosis. Ruling out lymphoproliferative disorders may also be very challenging before obtaining typical biopsy specimen. The first step is an assessment of epidemiological factors, notably the incidence of sarcoidosis and of alternative diagnoses; exposure to risk factors (e.g., infectious, occupational, and environmental agents); and exposure to drugs taken for therapeutic or recreational purposes. The clinical history, physical examination and, above all, chest computed tomography indicate which differential diagnoses are most likely, thereby guiding the choice of subsequent investigations (e.g., microbiological investigations, lymphocyte proliferation tests with metals, autoantibody assays, and genetic tests). The goal is to rule out all diagnoses other than sarcoidosis that are consistent with the clinical situation. Chest computed tomography findings, from common to rare and from typical to atypical, are described for sarcoidosis and the alternatives. The pathology of granulomas and associated lesions is discussed and diagnostically helpful stains specified. In some patients, the definite diagnosis may require the continuous gathering of information during follow-up. Diseases that often closely mimic sarcoidosis include chronic beryllium disease and drug-induced granulomatosis. Tuberculosis rarely resembles sarcoidosis but is a leading differential diagnosis in regions of high tuberculosis endemicity.

*How often have I said to you that when you have eliminated the impossible, whatever remains, however improbable, must be the truth?*

Sir Arthur Conan Doyle (Sherlock Holmes, *The Sign of Four*, 1890).

## 1. Introduction

Sarcoidosis is challenging to diagnose given the broad range of presentations, many of which mimic other conditions (1). No single criterion providing a definite diagnosis of sarcoidosis with 100% accuracy is available. Current guidelines indicate that the diagnosis of sarcoidosis should rely on three criteria, two positive (compatible presentation and evidence of noncaseating granulomas) and one negative (exclusion of all alternative diagnoses whose presentation and/or histopathology are similar) (1, 2). However, not all patients who meet these three criteria have sarcoidosis. The easiest criterion to assess is probably the presence of noncaseating granulomas, which can be sampled using highly efficient tools such as endobronchial ultrasound-guided transbronchial needle aspiration, transbronchial lung and bronchial biopsy, and biopsy of peripheral lesions (e.g., involving the skin or peripheral lymph nodes) (3). In contrast, assessing compatibility of the presentation and ruling out alternative diagnoses can be laborious. These two criteria must be evaluated concomitantly, since the presentation governs the likelihood of each alternative diagnosis. Determining whether the presentation is compatible with sarcoidosis relies heavily on expertise in the field of sarcoidosis. However, the diverse and nonspecific initial manifestations of sarcoidosis often lead patients to visit general practitioners or emergency physicians, who have limited experience with the disease. This point probably explains the often long diagnostic delays, which may exceed 6 months, and the need for several physician visits before the diagnosis is established (4).

Several recent articles provide useful information for diagnosing sarcoidosis. Development of the American Thoracic Society (ATS) diagnostic guidelines involved using a Delphi methodology to classify several manifestations as indicating a highly probable or probable diagnosis of sarcoidosis, based on the World Association of Sarcoidosis and Other Granulomatosis (WASOG) Sarcoidosis Organ Assessment Instrument (1, 5). Moreover, two Sarcoidosis Diagnostic Scores, with and without biopsy data, respectively, have been validated in confirmation cohorts, in the USA (6) and in a multicontinental study (7). The main strength of these studies is assessment of the scores under real-life conditions, with both sarcoidosis and a panel of alternative diseases mimicking sarcoidosis, to address both presentation compatibility and differential diagnoses.

This review focuses on the differential diagnosis of pulmonary sarcoidosis. Our aim was to provide practical diagnostic help to physicians who have limited experience with sarcoidosis. The first section presents useful diagnostic tools (epidemiology, clinical presentation, thoracic imaging, pathology, and microbiology) and provides examples of imaging and pathology findings. In the second section, we describe specific settings in which alternative diagnoses must be considered and discuss the best tools for differentiating these diagnoses from sarcoidosis.

## 2. Methods

We performed a comprehensive literature search for publications relevant to the differential diagnosis of sarcoidosis. To this end, we searched PubMed using the terms “sarcoidosis” OR “pulmonary granulomatosis” OR “tuberculosis” OR “histoplasmosis AND “epidemiology” OR “thoracic imaging” OR pathology” OR “microbiology” OR “occupational-induced” OR “environmental-induced” OR “drug-induced” OR “immunodeficiency” OR “genetic” OR “vasculitis” OR “Crohn’s disease.” We considered original articles and reviews published in English between January 2010 and January 2023, as well as other articles that did not meet these criteria but were of specific interest.

## 3. Results

### 3.1. How can epidemiology, clinical presentation and imaging, pathology, and microbiology studies help differentiate sarcoidosis from alternative diseases?

Table 1 lists the pulmonary granulomatous diseases to consider in the differential diagnosis of sarcoidosis, categorized according to their cause or mechanism. The table does not include granulomatous diseases that do not involve the lungs, such as cat scratch disease.

**Table 1:** Differential diagnoses of pulmonary granulomatous diseases that must be ruled out before diagnosing sarcoidosis.

Diseases	Clinical importance	Proportion of cases mimicking sarcoidosis (clinical and CT)	Circumstances and presentations
Infections			
Tuberculosis	++++	Low (multiple presentations)	TB-endemic countries; high individual risk
NTM infection	+	Low (only in rare presentations)	Individual risk; nodular pattern at imaging
Histoplasmosis	+	Low (only in rare presentations)	Living in or visiting endemic areas; nodular pattern or PH
Other infections	+/−	Very low	Vary across settings
Occupational/Environmental			
CBD	+++	High	Exposure history +++, few extrapulmonary manifestations
Other metal-induced granulomatosis	Very few data	Can mimic	Exposure Very rare
HP	+	Almost never	Causal antigen often identified
Hot-tub lung	+	Almost never (but pathology often similar)	Hot tub use
Granulomatous talcosis	+	High	Drug abuse (inhalation or intravenous injection)
Drug-induced granulomatosis			
TNF-a antagonists	+++	High	Treatments known to induce sarcoidosis-like reactions; with BCG therapy, the presentation can be different (miliary)
Checkpoint inhibitors			
Targeted therapies			
Intravesical BCG			
Interferons			
Other drugs			
Immunodeficiency			
G- CVID	+	Low	Recurrent infections, autoimmunity, hypogammaglobulinemia
CGD	+/−	Never	History of infections in infancy
Genetic disease			
Blau syndrome	+/−	Never	Onset before the 3–4 years of age; no lung involvement; familial history in 40%
Vasculitis, CTD, inflammatory disease			
GPA	+	Rare (in particular presentations)	multiple consolidation; nasosinusal manifestations
EGPA	−	Never	Asthma; eosinophilia
NSG	+++	High	Main issue is nosological
ILD in Sjögren’s syndrome	+	Rare	In exceptional cases, sarcoidosis mimics NSIP
Crohn’s disease	+	Almost never	Consider drug-induced granulomatosis or association with sarcoidosis
Proliferative disorders			
Cancer	++	Rare (in particular presentations)	Lymphadenopathy and lung nodules
Lymphoma	++	Rare (with an expert radiologist)	Possible association or succession of sarcoidosis and lymphoma
Lymphomatoid granulomatosis	+	Rare (in particular presentations)	Lung nodules

NTM: nontuberculous mycobacteria; TB: tuberculosis; PH: pulmonary hypertension; CBD: chronic beryllium disease; HP: hypersensitivity pneumonitis; G-CVID: granulomatosis-associated common variable immune deficiency; CGD: chronic granulomatous disease; CTD: connective tissue disease; GPA: granulomatosis with polyangiitis; EGPA: eosinophilic granulomatosis with polyangiitis; NSG: necrotizing sarcoid granulomatosis; ILD: interstitial lung disease; NSIP: nonspecific interstitial pneumonia.

#### 3.1.1. Ratio of incidences of sarcoidosis over alternative diagnoses according to the epidemiological setting

The ratio of the incidence of sarcoidosis over the incidence of each alternative diagnosis varies significantly across geographic areas and across sub-populations in a given area. These variations dramatically impact the probabilities of different diagnoses, according to Bayes’ theorem. A comparison of the incidences of sarcoidosis and tuberculosis illustrates this point. In some countries, tuberculosis is the main differential diagnosis of sarcoidosis (8, 9). Sweden has a high incidence of sarcoidosis (11.5/100,000/y) (10) and a very low incidence of tuberculosis (3.6/100,000/y), Italy a low incidence of sarcoidosis (1.2–3/100,000/y) and higher incidence of tuberculosis (6.6/100,000/y), and Russia a low incidence of sarcoidosis (1.1–3.8/100,000/y) and high incidence of tuberculosis (46/100,000/y). The probability that a patient will have sarcoidosis rather than tuberculosis therefore varies widely across these three countries, the incidence ratios being 3.2 in Sweden, 0.18 in Italy, and 0.02 in Russia; the value in Russia is 160 times smaller than that in Sweden. In some countries, both diseases are very common: in India, the prevalence of sarcoidosis is 61.2–150/100,000 (incidence unknown) and the incidence of tuberculosis is 188/100,000/y. As discussed below, the diagnosis of sarcoidosis is considerably more difficult in regions where tuberculosis is endemic. Moreover, in a given area, significant epidemiological differences may exist across ethnic groups, as shown by a study done in a Paris suburb, where the odds ratio for having sarcoidosis was 2.97 in black people from the Caribbean or subsaharian Africa, with European Caucasians as the reference (11). Similarly, in high-income countries, tuberculosis is far more common in migrants from low-income countries than in locals.

Contrary to tuberculosis, pulmonary histoplasmosis is not ubiquitous but instead occurs in well-delineated areas such as the Ohio River and Mississippi River valleys in the USA and specific regions of Central and South America, Africa, Asia, and Australia. This disease is very rare in Europe, with the exception of Italy (12). Patients must be asked about travel to endemic areas. In the USA, nontuberculous mycobacterial infections are more common than tuberculosis and must be included in the differential diagnosis (13). In a biopsy study comparing the causes of pulmonary granulomatosis in the USA and in other countries (Austria, Brazil, India, Japan, Scotland, and Turkey), pulmonary histoplasmosis was demonstrated in 18 patients in the USA vs. none in the other countries, whereas tuberculosis was found in a single patient in the USA vs. 18 in the other countries (14). Thus, region-specific epidemiological factors weigh heavily on the differential diagnosis.

Chronic beryllium disease and other occupational mineral-induced granulomatous diseases, hypersensitivity pneumonitis, and drug-induced granulomatosis occur in specific settings. Blau syndrome, which very rarely involves the lungs, is usually diagnosed at a younger age compared to pediatric sarcoidosis (15).

#### 3.1.2. Clinical presentation

Symptoms and signs, particularly those observed in extra-pulmonary manifestations which may be observed in up to 50% of sarcoidosis patients, may be useful to differentiate sarcoidosis from alternative diseases. For example, some manifestations were classified supportive of sarcoidosis diagnosis with a high probability (erythema nodosum, lupus pernio or uveitis) or with probability (seven cranial nerve paralysis) according to official American Thoracic Society Clinical Practice Guidelines (1) Recently, a Sarcoidosis Diagnosis Score Clinical could be validated in a large multicontinental sarcoidosis population compared to controls with multiple other diseases made possible to assess the probability of sarcoidosis diagnosis before obtaining typical granulomas on biopsy specimens (7).

#### 3.1.3. Thoracic imaging

The likelihood of specific findings (e.g., lymphadenopathy, lung infiltration with or without fibrosis, nodules, pulmonary hypertension) differs between sarcoidosis and the alternative diagnoses.

##### 3.1.3.1. Hilar and mediastinal lymphadenopathy

**Figure 1:**
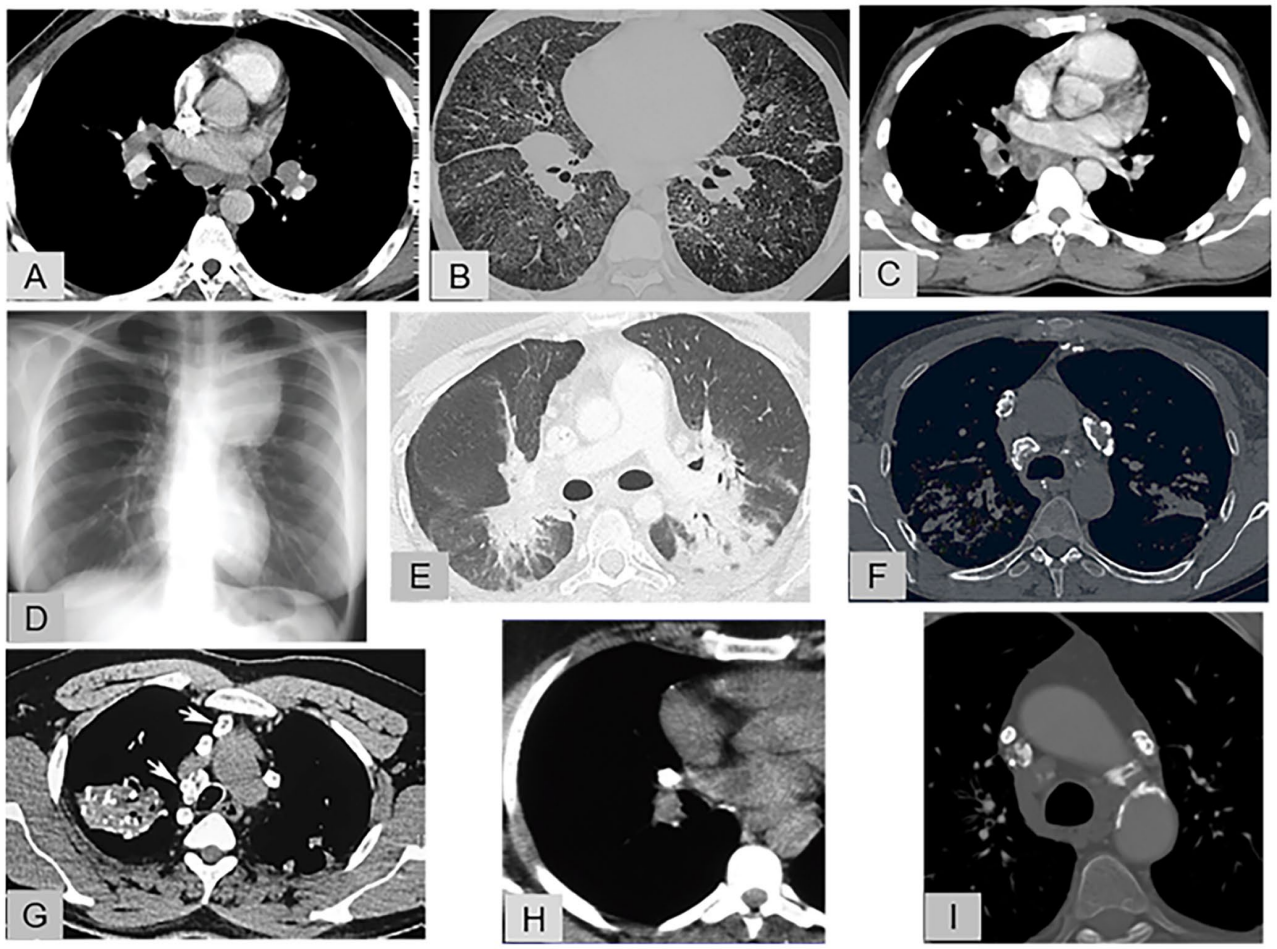
Representative features of lymphadenopathy. **(A)** Enhanced chest CT (mediastinal window): Sarcoidosis with typical, symmetric, hilar and mediastinal lymphadenopathy that does not compress the adjacent pulmonary vessels, **(B)** Chest CT (pulmonary window): Sarcoidosis with a rare and atypical lymphadenopathy pattern combining unilateral hilar and mediastinal lymphadenopathy, thickened fissures, diffuse ground-glass opacities, and sparse parenchymal micronodules. **(C)** Enhanced chest CT (mediastinal window): Tuberculosis with unilateral hilar and mediastinal lymphadenopathy exhibiting central necrosis. **(D)** Chest radiography: Hodgkin’s disease with voluminous, left, anterosuperior, mediastinal lymphadenopathy. **(E)** Enhanced chest CT (pulmonary window): Immune checkpoint inhibitor-induced sarcoid-like reaction: bilateral hilar lymphadenopathy with blurred contours extending as ground-glass opacities into the posterior lung parenchyma. **(F)** Chest CT (mediastinal window): Sarcoidosis: mediastinal lymphadenopathy with eggshell calcifications. **(G)** Chest CT (mediastinal window): Silicosis: asymmetric, calcified, mediastinal lymphadenopathy, with eggshell calcification of some nodes; calcified mass in the right upper lung lobe and small, scattered calcifications in the left lung. **(H)** Chest CT (mediastinal window): Tuberculosis: completely calcified unilateral hilar lymph node. **(I)** Chest CT (mediastinal window): Amyloidosis: small, bilateral, calcified, mediastinal lymphadenopathy.

Figures 1A–I shows representative features of lymphadenopathy by chest computed tomography (CT), from the most typical to the most atypical, in sarcoidosis and in the alternative diagnoses.

###### 3.1.3.1.1. Bilateral hilar lymphadenopathy with or without mediastinal lymphadenopathy

In 70% of patients with sarcoidosis, chest radiographs show typical lymphadenopathy, with or without pulmonary infiltrates. Parenchymal disease is evident in only 45% of patients at the time of maximal lymphadenopathy. In patients without parenchymal infiltrates, the lymphadenopathy is hilar and bilateral (98%). In a study of 62 patients, the most common mediastinal locations were the aortopulmonary (76%) and right paratracheal (71%) regions and the most common combination was bilateral hilar, right paratracheal, and aortopulmonary (37%) (16). Symmetrical hilar lymphadenopathy is a characteristic finding (Figure 1A) and can help distinguish sarcoidosis from other conditions responsible for hilar and mediastinal lymphadenopathy (17). Typically, sarcoid lymphadenopathy does not compress the adjacent airways and vessels.

Other conditions whose features can include symmetrical bilateral hilar lymphadenopathy are chronic beryllium disease (*cf infra*) in which the node enlargement is usually moderate, and drug-induced granulomatosis (18, 19) (Figure 1E).

By contrast, symmetrical bilateral hilar lymphadenopathy was a presentation feature in only 3.8% of lymphomas, 0.8% of bronchogenic carcinomas, and 0.2% of extrathoracic carcinomas (20). In granulomatosis-associated common variable immunodeficiency, bilateral hilar lymphadenopathy indistinguishable from sarcoidosis was seen in only 10% of patients (21).

In IgG4-related disease, the chest CT presentation very rarely suggests sarcoidosis, with lymphadenopathy, peribronchovascular thickening, and nodules. The risk of confusion is greatest in patients who have symmetrical swelling of the lacrimal, parotid, and submandibular glands. However, findings of auto-immune pancreatitis, bilateral orbital pseudo-tumor, and retroperitoneal fibrosis with no granulomas but typical features of IgG4-related disease upon histological examination provide the correct diagnosis (22, 23).

###### 3.1.3.1.2. Unilateral or asymmetrical hilar lymphadenopathy, or mediastinal lymphadenopathy without hilar lymphadenopathy

Asymmetric or unilateral hilar or mediastinal lymphadenopathy occurs in less than 5% of patients with sarcoidosis overall (24) but is more common after 50 years of age (Figure 1B) (24).

Unilateral hilar lymphadenopathy is more often seen in tuberculosis, histoplasmosis, lymphoma, bronchogenic carcinoma, and metastatic carcinoma than in sarcoidosis (25).

Tuberculosis must be considered routinely (26). Features that strongly suggest active tuberculosis include unilateral hilar/paratracheal lymphadenopathy (Figure 1C), mediastinal lymphadenopathy with peripheral rim enhancement (due to central necrosis) or heterogeneous enhancement (Figure 1C), and lymph-node conglomeration or obscuration of perinodal fat with or without ipsilateral parenchymal lesions.

In the acute form of pulmonary histoplasmosis in immunocompetent patients, lymphadenopathy may be present (20% of patients), and small nodules (<3 mm) are usually seen (80% of patients) (27).

Large lymphadenopathy in the anterosuperior mediastinum suggests lymphoma (Figure 1D) (20).

###### 3.1.3.1.3. Calcified lymph nodes

Eggshell calcifications can be seen in sarcoidosis (Figure 1F), some fungal infections, and amyloidosis but are more common in silicosis (Figure 1G) and coal miner’s pneumoconiosis (28). In a retrospective CT study, nodal calcifications were present in 53% of 49 patients with sarcoidosis and 46% of 28 patients with tuberculosis (29). Focal calcification was more common in sarcoidosis (58%) than in tuberculosis (23%), whereas the opposite was true for complete calcification (62 and 27%, respectively) (Figure 1H). When the nodes were calcified, involvement was far more often bilateral in sarcoidosis than in tuberculosis (65 and 8%, respectively) (Figures 1F,H) (29). In patients with systemic amyloidosis, lymphadenopathy was the single most common abnormality (75%) and contained punctate calcifications in 33% of cases (Figure 1I).

##### 3.1.3.2. Lung involvement

###### 3.1.3.2.1. Micronodular parenchymal pattern

**Figure 2:**
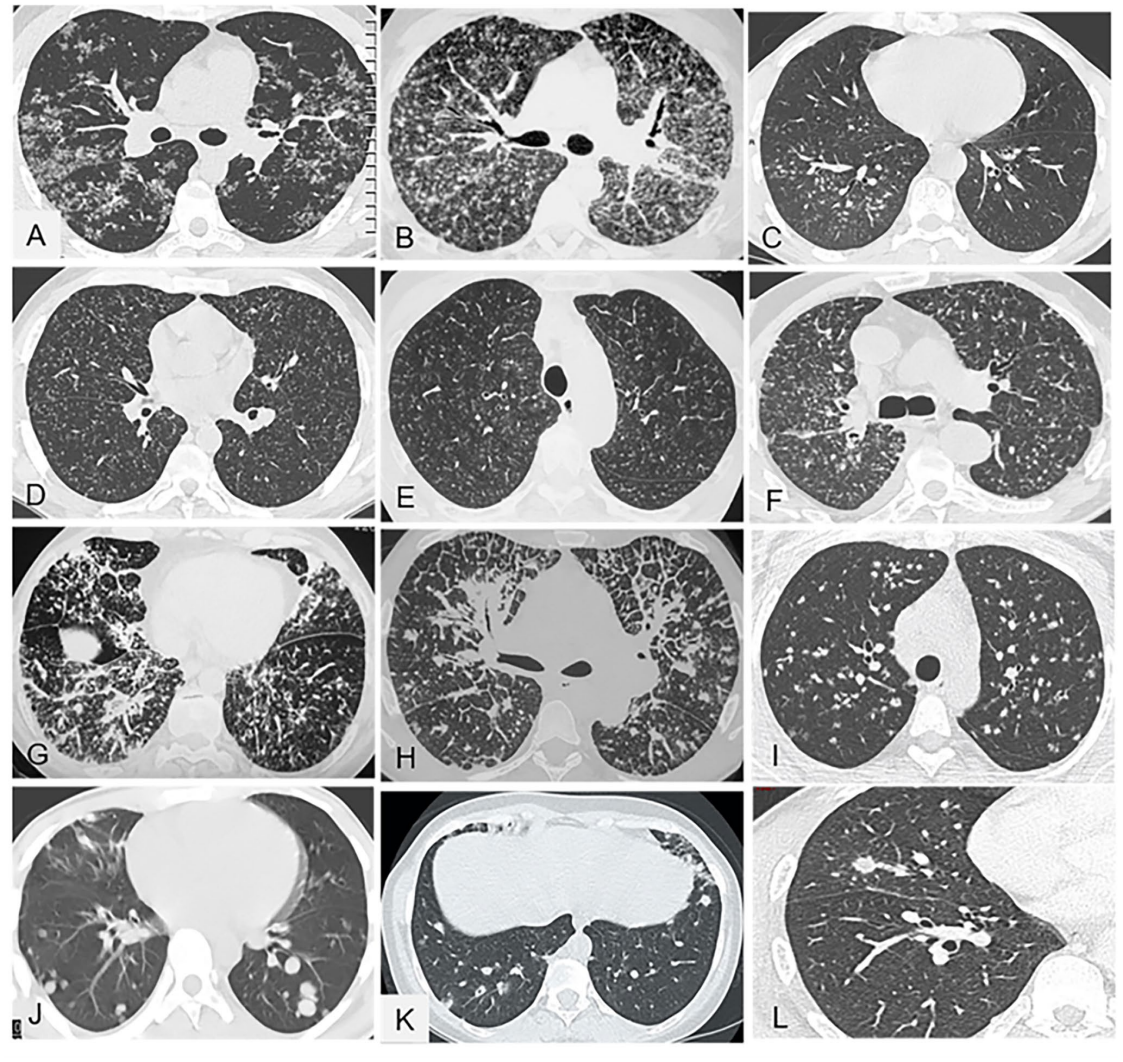
Representative of micronodular parenchymal pattern (A-H) and nodular pattern (I-L) in chest CT (parenchymal window). **(A)** Sarcoidosis with typical diffuse micronodules aggregated in small clusters and predominating along the fissures (lymphatic distribution). **(B)** Sarcoidosis: less typical presentation with profuse micronodules whose distribution is more difficult to determine; nevertheless, the predominance of micronodules along the fissures is suggestive of lymphatic distribution. **(C)** Bronchogenic tuberculosis with micronodules in a unilateral and centrilobular distribution that spares the pleural surface. **(D)** Typical, hematogenous, miliary tuberculosis with minute micronodules that are all the same size and are distributed uniformly (i.e., at random) throughout both lungs. **(E)** Hypersensitivity pneumonitis with profuse, minute, diffuse micronodules in a centrilobular distribution that spares the subpleural parenchyma, leaving a 2–3 cm uninvolved border. **(F)** Neoplastic miliary: the distribution is not symmetrical and the nodules are larger and more irregular than in miliary tuberculosis. **(G)** Systemic amyloidosis with diffuse pulmonary involvement and no lymphadenopathy; the micronodules are diffuse with no particular distribution and are combined with several irregular lines and thickened interlobular septa. **(H)** Lymphangitis carcinomatosa: thickened peribronchovascular interstitium, nodules, and nodular septal reticulations without distortion. **(I)** Pulmonary sarcoidosis: multiple nodules with irregular and well-defined contours. **(J)** Pulmonary metastases from renal carcinoma: sharply contoured nodules in a more peripheral distribution. **(K)** Granulomatosis-associated common variable immunodeficiency: nodules, some of which exhibit the halo sign. **(L)** Chronic histoplasmosis: sparse pulmonary nodules.

Figures 2A–H illustrates this pattern.

The typical radiographic manifestation of parenchymal lung involvement by sarcoidosis consists in diffuse micronodules predominating in the upper and middle parts of the lungs (16).

By chest CT, perilymphatic micronodules are the most common abnormality in pulmonary sarcoidosis (77% of cases) (30). These opacities predominate in the upper and middle lungs (68%). Clusters of micronodules and nodules are often visible around the peripheral bronchovascular bundles (Figure 2A) (30). The micronodules may be so profuse as to make their distribution difficult to assess. However, micronodule predominance along the fissures suggests a perilymphatic distribution (Figure 2B). At the level of the secondary pulmonary lobules, the interlobular septa are thickened or nodular and the centrilobular interstitium is thickened (31). In a study of pulmonary sarcoidosis, 15 of the 25 patients had nodular lesions 1 to 5 mm in diameter (32). These nodules predominated along the bronchovascular bundles in 17 patients and, to a lesser extent, in the subpleural regions in 19 patients and along the interlobular septa, with a beaded appearance. Thickening of the interlobular septa was seen in 10 patients. The nodule contours were irregular in 17 patients.

**Figure 3:**
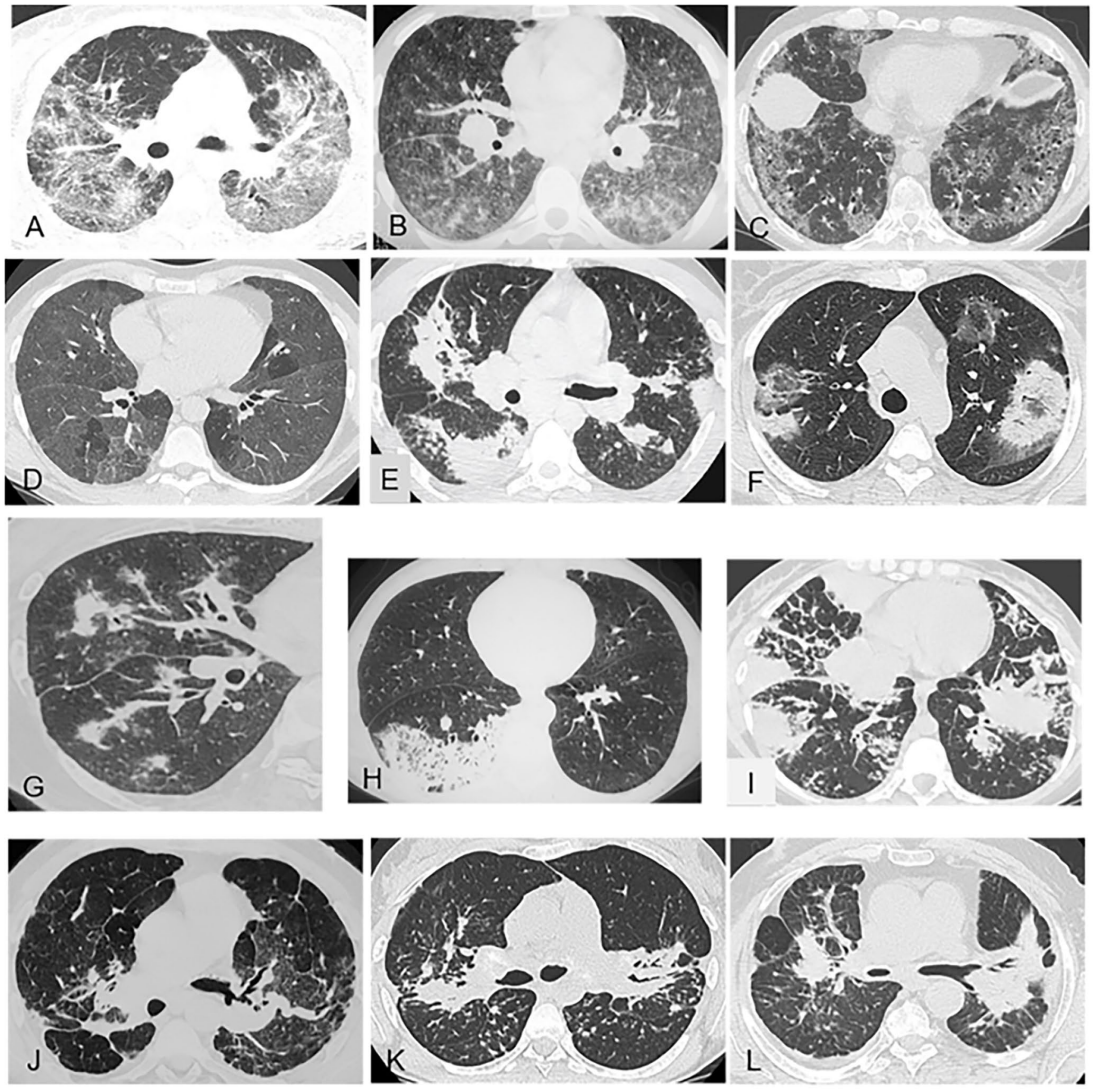
Representative of gound-glass opacities **(A–D)** and alveolar consolidations **(E–I)** and masses **(J–L)** on chest CT (pulmonary window). **(A)** Pulmonary sarcoidosis with predominant, diffuse, ground-glass opacities combined with lymphatic micronodules. **(B)** Chronic beryllium disease: heterogeneous ground-glass opacities with bilateral hilar and mediastinal lymphadenopathy. **(C)** Nonspecific interstitial pneumonia: diffuse ground-glass opacities and fine reticulations sparing the subpleural lung. **(D)** Hypersensitivity pneumonitis: diffuse ground-glass opacities and multiple clear lobules trapped in expiration. **(E)** Pulmonary sarcoidosis with bilateral alveolar consolidations and lymphatic micronodules. **(F)** Cryptogenic organizing pneumonia: bilateral peripheral consolidations with lower-density (ground-glass) centers. **(G)** Granulomatosis with polyangiitis: alveolar consolidation related to a pulmonary vessel. **(H)** Lepidic-growth carcinoma: pneumonic form with a unilateral focus. **(I)** Lymphoma: bilateral alveolar consolidations with a limited number of septal reticulations and micronodules. **(J)** Fibrosing pulmonary sarcoidosis: typical presentation with bilateral hilar masses and bronchial distortion. **(K)** Sarcoidosis with bilateral masses in the posterior segments of the upper lobes and micronodules. **(L)** Coal miner’s pneumoconiosis: bilateral mass-like lesions in the upper lobes.

A limited number of sarcoidosis-like granulomatous diseases can closely mimic sarcoidosis, including chronic beryllium disease and drug-induced granulomatous diseases (19, 33). In 28 patients with chronic beryllium disease, the chest CT findings were similar to those reported in sarcoidosis, with nodules predominating in the peribronchovascular regions or along the interlobular septa (57%), interlobular septal thickening (50%), hilar and mediastinal lymphadenopathy (39%), and other findings such as ground-glass opacities (32%) (Figure 3B) (33).

Granulomatosis induced by checkpoint-inhibitor cancer immunotherapy is becoming increasingly common due to the expanding indications for these drugs (34). The imaging features resemble those of sarcoidosis, with perilymphatic nodules predominating in the upper lungs and with mediastinal and hilar lymphadenopathy (35). The immune checkpoint inhibitor most often associated with a sarcoid-like reaction is ipilimumab. Melanoma was initially the most common underlying malignancy (34). The main other drugs that can induce sarcoid-like reactions (TNFa antagonists and biosimilars, interferons and pegylated interferons, and targeted therapies against cancer) can also produce chest CT lung abnormalities mimicking sarcoidosis.

By contrast, in tuberculosis, the most typical lesions in the event of airway dissemination are nodules, centrilobular nodules (notably tree-in-bud nodules), and clustered nodules predominating in the upper lobes, right middle lobe, lingula, and superior segment of the lower lobes (Figure 2C); and consolidation in these same regions with ipsilateral lymph node enlargement. Hematogenous dissemination produces a typical miliary pattern (Figure 2D); thick-walled cavities; cavities with surrounding consolidation, especially in the upper and middle parts of the lungs; pleural effusion; and the split pleura sign with separation of the two pleural leaflets by an effusion or empyema (26).

The most common radiographic abnormality in acute symptomatic histoplasmosis consists of multiple nodular opacities usually smaller than 3 mm in diameter, generally in a diffuse distribution (36). Pleural effusions are rare (2%).

In hypersensitivity pneumonitis, profuse, minute, diffuse micronodules in a centrilobular distribution sparing the subpleural parenchyma are frequently described (Figure 2E).

Several nongranulomatous lung diseases may mimic sarcoidosis, such as silicosis and coal miner’s pneumoconiosis, in which micronodules predominate in the upper lungs (78%), in a centrilobular and subpleural, bilateral, and symmetrical distribution, sometimes with confluence and posterior predominance (38%) (Figure 3L) (37, 38). Granulomatous talcosis is a pulmonary disease secondary to inhaled or intravenous drug abuse. Diffuse bronchiolar micronodular lesions are visible, sometimes in combination with masses containing areas of amorphous density (39).

In lymphangitis carcinomatosa, the nodules share with sarcoidosis a predominance in the peribronchovascular regions (11 of 18 cases) (40). However, nodular polygonal lines (51%) or thickened undistorted septal lines (66%), pleural fluid (60%), and unilateral predominance of small opacities (38%) strongly suggest lymphangitis carcinomatosa (Figure 2H) (40). Thus, the predominant lesions often differ from those found in sarcoidosis (41). The beaded septum sign consisting in nodular thickening of the interlobular septa is common in lymphangitis carcinomatosa and less so in sarcoidosis (42). In neoplastic miliary, the nodules are not symmetrically distributed and are larger and more irregular than in miliary tuberculosis (Figure 2F).

Pulmonary amyloidosis may produce misleading imaging-study findings such as diffuse micronodules in a predominantly perilymphatic distribution (41, 43, 44). In a retrospective study, 12 of 19 patients with proven amyloidosis had the systemic form of the disease, including six with both lymphadenopathy and diffuse parenchymal involvement and two with only the latter manifestation (44). Of the eight patients with diffuse parenchymal involvement, six had pulmonary nodules (Figure 2G), four diffuse irregular lines or interlobular septal thickening, three honeycombing, and three patchy ground-glass opacities.

###### 3.1.3.2.2. Less typical patterns of lung infiltration in sarcoidosis

**Figure 4:**
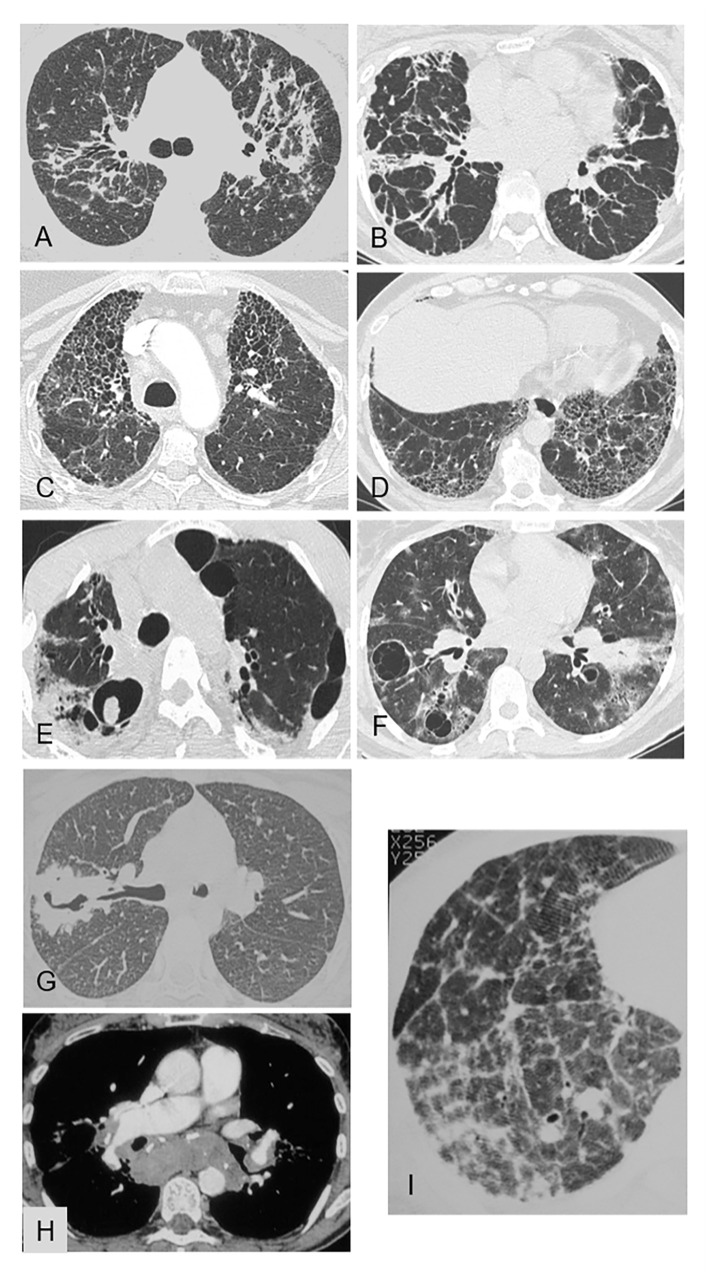
Representative of linear opacities **(A,B)**, honeycombing **(C,D)** cysts and cavities **(E–G)** pulmonary hypertension **(H,I)** on chest CT [pulmonary window **(A–G; I)** and mediastinal window **(H)**]. **(A)** Pulmonary sarcoidosis: bilateral linear opacities extending from the hilum to the subpleural area, with distortion. **(B)** Nonspecific interstitial pneumonia: bilateral linear opacities extending from the hilum to the subpleural area, with distortion. **(C)** Fibrosing pulmonary sarcoidosis: honeycombing with joined cavities predominating in the upper lobes. **(D)** Usual interstitial pattern in idiopathic pulmonary fibrosis: honeycombing with joined cavities that are located in the lower lobes in contact with the pleura and are smaller than in sarcoidosis. **(E)** Sarcoidosis with chronic pulmonary aspergillosis: fibrotic lung lesions with cavities and an aspergilloma within a right upper-lobe cavity. **(F)** Granulomatosis-associated common variable immunodeficiency: thin-walled cystic air spaces, patchy areas of ground-glass opacities in both lungs, thickened peribronchovascular interstitium, and bilateral hilar lymphadenopathy. **(G)** Granulomatosis with polyangiitis: consolidation with a thick-walled cavity in the right upper lobe. **(H)** Sarcoidosis with pulmonary hypertension: compression of the right branch of the pulmonary artery by lymphadenopathy. **(I)** Sarcoidosis with pulmonary hypertension: septal reticulation and distortion; note that the pulmonary arteries are wider than the bronchi.

These patterns are illustrated in Figures 3, 4.

In about 10% of patients with sarcoidosis, the predominant chest CT finding consists in ground-glass opacities (Figure 3A) or alveolar consolidations (Figure 3E).

**Predominant ground-glass** opacities in patients without immunodepression occur chiefly in connective tissue diseases, drug-induced pneumonitis, alveolar proteinosis, nonspecific interstitial pneumonia (Figure 3C), and smoking-related diseases. However, the main differential diagnosis with sarcoidosis is hypersensitivity pneumonitis, in which the ground-glass opacities are usually extensive or combined with other findings consisting chiefly in centrilobular micronodules with no tree-in-bud appearance nor involvement of the pleura or fissures (Figure 3D). The opacities are diffuse and symmetrically distributed. Their contours are blurred and their density usually low. Other signs of hypersensitivity pneumonitis are air trapping, the three-density pattern, and fibrosis. Foci of lobular air trapping on expiratory scans have been reported to be common (Figure 3D). Cystic cavities are visible in a minority (15%) of patients with hypersensitivity pneumonitis. Of note, although predominant ground-glass opacities are rare in sarcoidosis, a study demonstrated that the logical analysis of CT data established the diagnosis of sarcoidosis with high accuracy in patients with predominant ground-glass lesions, due to the additional presence of either lymphadenopathy in 79% of cases or fissural micronodules in 55% of cases (45).

Predominant multiple **consolidations** are more common in organizing pneumonia (Figure 3F), granulomatosis with polyangiitis (Figure 3G), lymphoma (Figure 3I), and lepidic-growth carcinomas (Figure 3H) than in sarcoidosis. However, as with ground-glass opacities, co-existing abnormalities can help to establish the diagnosis of sarcoidosis (46).

###### 3.1.3.2.3. Nodules

Sarcoidosis can also manifest as multiple nodules (defined as >3 mm in diameter) (Figure 2I). Alternative diagnoses include other granulomatous diseases, notably infections [e.g., tuberculosis, nontuberculous mycobacterial infections, and histoplasmosis (Figure 2L)], drug-induced granulomatosis, granulomatosis with polyangiitis, and eosinophilic granulomatosis with polyangiitis. In granulomatosis-associated common variable immunodeficiency (Figure 2K), chest CT findings seen more often than in sarcoidosis were nodules, which were sometimes multiple, with the halo sign (30% vs. 3.5%) and smooth margins; air bronchograms; and bronchiectasis (65% vs. 23%) (21). The distribution of micronodules was perilymphatic in 42% of patients with granulomatosis-associated common variable immunodeficiency vs. 100% of patients with sarcoidosis. Nongranulomatous diseases such as metastases (Figure 2J), lymphoma, lymphoid granulomatosis, and amyloidosis should also be considered.

###### 3.1.3.2.4. Cysts and other cavities

True primary pulmonary sarcoid cavitation, in which the walls of the cavities are formed by characteristic noncaseating lesions, has been reported in 2.2% of patients with sarcoidosis (47). However, even in patients with sarcoidosis, cavitation is more often due to necrotizing pyogenic, mycobacterial, mycotic, or parasitic infections (Figure 4E) (25).

Other interstitial lung diseases can cause cavities. Thus, thin-walled cystic air-filled spaces can develop in lymphocytic interstitial pneumonia (Figure 4F) and thick-walled cavities in granulomatosis with polyangiitis (Figure 4G).

##### 3.1.3.3. Pulmonary fibrosis

Figures 3, 4 show the chest CT features of pulmonary fibrosis.

###### 3.1.3.3.1. Progressive massive fibrosis

Progressive massive fibrosis often manifests as mass-like lesions, usually in a bilateral upper-lobe distribution, not only in sarcoidosis (Figures 3J,K) but also in silicosis dusts (Figure 3L), coal miner’s pneumoconiosis with heavy exposure to inorganic dusts, and granulomatous talcosis (48, 49). Background nodular opacities are associated with pneumoconiosis, with or without emphysematous destruction adjacent to the massive fibrosis (48).

###### 3.1.3.3.2. Irregular septal thickening and irregular linear opacities from hilum to subpleural lung

A common chest CT finding in sarcoidosis is interlobular septal thickening, which is often irregular or associated with marked distortion of the lung structures (49). Linear opacities extending from the hilum to the lung periphery, with distortion, are also common in sarcoidosis (Figure 4A) and are much rarer in nonspecific interstitial pneumonia (Figure 4B).

Several signs of fibrosis strongly suggest sarcoidosis. In a study of 27 patients with sarcoidosis, nine patients exhibited varying degrees of parenchymal distortion consistent with fibrosis (50) and in another, lobular distortion was a feature in 13 of 25 patients with sarcoidosis (32).

###### 3.1.3.3.3. Honeycombing

Fibrosing pulmonary sarcoidosis can produce honeycomb cavities (Figure 4C). Honeycombing predominates in the upper lobes, and the cavities are usually large. In usual interstitial pneumonia, the joined cavities are smaller than in sarcoidosis and located in the lower lobes in contact with the pleura (Figure 4D).

##### 3.1.3.4. Pulmonary hypertension

In pulmonary hypertension, signs of lobular distortion (Figure 4I), fibrosing mediastinitis, and pulmonary-artery compression by lymph nodes (Figure 4H) help to suggest sarcoidosis as the cause (51). When these signs are absent and the disease is recognized very late, it may be extremely difficult to eliminate other causes of pulmonary hypertension such as histoplasmosis or nongranulomatous diseases, notably interstitial lung disease-associated connective vascular diseases (52).

#### 3.1.4. Pathology

**Figure 5:**
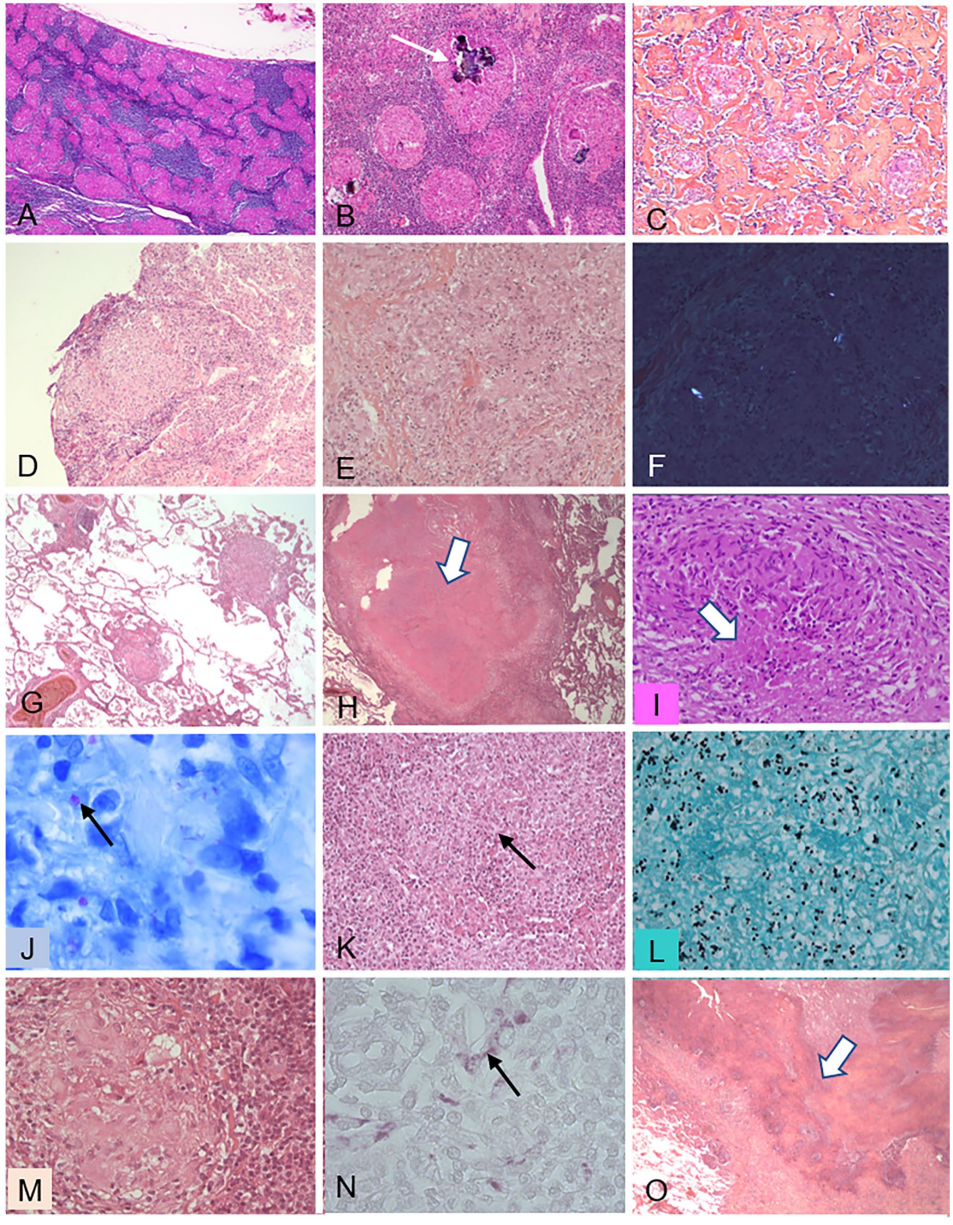
Histopathological features of granulomas in various diseases. This figure illustrates several features of granulomas observed in various granulomatous diseases, either in mediastinal lymph nodes **(A–C)** or in pulmonary biopsies **(D–O)**. **(A–C)** Sarcoidosis. **(A)** (X50): nonnecrotizing granulomas filling most of the node tissue; **(B)** (X100): Schaumann’s bodies (arrow); **(C)** (X100): dense fibrosis intermingled with granulomas. **(D)** Chronic beryllium disease: nonnecrotizing granuloma in a transbronchial biopsy (X200). **(E,F)** Intravenous pulmonary talcosis. **(E)** (X300): granuloma mimicking sarcoidosis; F (X300) birefringent talc particles visible under polarized light. **(G–J)** Tuberculosis. **(G)** (X50): low-magnification view showing the perivascular distribution of granulomas in miliary tuberculosis; **(H)** (X50): large nodule composed of caseating necrosis (arrow); **(I)** (X300): granuloma with a limited focus of necrosis (arrow); **(J)** (X950): Ziehl-Neelsen stain showing tuberculous mycobacteria (arrow). **(K,L)** Histoplasmosis. **(K)** (X300): granuloma with a central necrotic focus (arrow); **(L)** (X500): Grocott stain showing numerous yeast forms of *Histoplasma capsulatum.* **(M,N)** Whipple’s disease. **(M)** (X300): granuloma with foamy histiocytes; **(N)** (X500): periodic-acid Schiff-positive inclusions within the foamy histiocytes. **(O)** Granulomatosis with polyangiitis (GPA): low-magnification view (X50) showing geographic basophilic necrosis suggestive of GPA when observed within a granulomatous pneumonitis.

A careful examination of biopsy specimens can help with the differential diagnosis, since several microscopic features differ between sarcoidosis and the alternative diseases. We highlight the most typical and most atypical findings in sarcoidosis and in most alternative diseases which are summarized in Table 2. Figure 5 illustrates several histopathological findings.

**Table 2:** Histological patterns of granulomatous interstitial lung diseases.

Diseases	Main granuloma patterns	Main sites involved	Additional features	Special methods for causal-agent identification
	Sarcoidosis			
Sarcoidosis	Well-circumscribed, coalescent, epithelioid and giant-cell, noncaseating granulomas surrounded by lymphocytes, in a peripheral envelope of lamellar fibrosis Very rarely, minimal focal necrosis Granulomatous vasculitis	Mediastinal lymph nodes In the lung: - Along the lymphatics - Usually along the pulmonary vessels, lobular septa, visceral pleura, and large and small airways. - Granulomatous vasculitis predominantly involving the small pulmonary veins	Nonspecific cytoplasmic inclusions mostly in multinucleate giant cells: - Schaumann and crystalline bodies (iron and calcium salts) - Asteroid bodies (cholesterol) Usually with scant pulmonary inflammation Confluent granulomas with necrosis (NSG pattern)	None, except special staining or polarized light microscopy to exclude agents involved in other granulomatous diseases (minerals, mycobacteria, fungi).
	Most common granulomatous lung diseases that must be distinguished from sarcoidosis			
Chronic beryllium disease (CBD)	Granulomas mimicking sarcoidosis: well circumscribed, often coalescent granulomas, combined with poorly formed granulomas Fibrosis at the periphery of granulomas, coalescing into hyalinizing nodules	Mediastinal lymph nodes Lymphangitic, frequently associated with scattered small lobular granulomas	Interstitial fibrosis Deposits of various dusts depending on associated respiratory exposures Residual Schaumann bodies	None
Drugs	Granulomas mimicking sarcoidosis	Mediastinal lymph nodes Airway mucosa Pulmonary parenchyma		None
Tuberculosis (TB) (*Mycobacterium tuberculosis* infection)	Early stages: necrotizing granulomas containing mycobacteria; neutrophils Interstitial caseating and noncaseating granulomas At the late stage, palisades of epithelioid histiocytes delineating geographic-shaped necrosis, cavitation, rare neutrophils Little or no fibrosis Granulomas are less well organized in immunocompromised patients	Mediastinal lymph nodes Aerogenic TB: early lesions centered on the bronchioles; later, randomly distributed lesions Hematogenous miliary TB: multiple scattered nodules made of groups of granulomas	Frequently, areas of organizing pneumonia Alveolar and interstitial acute inflammation Secondary vasculitis	Ziehl-Neelsen stain or fluorochrome stain using auramine O Special cultures PCR
Hypersensitivity pneumonitis (subacute)	Small and poorly structured nonnecrotizing noncoalescent granulomas Schaumann bodies Fibrosis at the periphery of granulomas	Airway-centered inflammation (bronchioles and alveolar ducts) Bronchiolocentric interstitial pneumonitis with sparse, poorly structured granulomas	Interstitial infiltration by lymphocytes with lymphoid aggregates Frequently, areas of bronchiolitis obliterans and organizing pneumonia Lymphoid follicles	None
Fungal infection by *Histoplasma capsulatum*	Transition from the acute phase: granulomas with central areas of necrosis replace the mononuclear infiltrate Chronic phase: - noncaseating well-structured granulomas (similar to sarcoidosis); - necrotizing granulomas; - concentric lamellar fibrosis (similar to reactivated TB)	Mediastinal lymph nodes Lung parenchyma Rarely, bronchocentric granulomatosis	Acute phase: acute fibrinous pneumonia without granulomas Pulmonary fibrosis Sclerosing mediastinitis Calcification of nodules	PAS Grocott methenamine silver stain
Pulmonary granulomatous-associated common variable immunodeficiency (CVID/GD)	Granulomatous and lymphocytic interstitial lung disease Clusters of alveolar and interstitial nonnecrotizing granulomas admixed with lymphocytic interstitial pneumonia	Mediastinal lymph nodes Peribronchiolar	Organizing pneumonia Lymphoid interstitial pneumonia/ Lymphoid hyperplasia Follicular bronchiolitis	
Granulomatosis with polyangiitis (GPA)	Necrosis and granulomatous vasculitis Poorly structured granulomas; clusters of giant cells; small suppurative granulomas Granulomas within the vessel wall Palisading granulomas	Focal granulomatous vasculitis affecting medium-sized and small veins and arteries Loosely scattered multinucleated giant cells	Association: - Parenchymal geographic basophilic necrosis - Vasculitis, endotheliolitis, capillaritis - Granulomatous inflammation with neutrophilic micro-abscesses - Collagen necrosis	
Eosinophilic granulomatosis with polyangiitis (EGPA)	Necrotizing vasculitis with fibrinoid necrosis Necrotizing granulomas with eosinophils in the center Eosinophilic inflammation		Eosinophilic pneumonia Eosinophilic small-vessel vasculitis (small arteries, veins, and capillaries)	
	Less common and rare granulomatous lung diseases that must be distinguished from sarcoidosis			
Granulomatous pneumonitis due to inorganic agents	- Aluminum: pseudo-sarcoid granulomas - Hard metal: giant-cell interstitial pneumonia, infarcted granulomas	- Aluminum: occasional scattered sarcoid-like granulomas - Hard metal: centrilobular nodules	-Aluminum: centri-acinar macules - Hard metal: Numerous multinucleate giant cells; diffuse giant-cell alveolitis	Scanning Electron Microscopy/Energy Dispersive X-ray Spectroscopy (SEM/EDS)
Intravenous drug abuse-related lung disease (intravascular talcosis)	Interstitial foreign-body granulomas Granulomatous vasculitis	Juxtavascular and perivascular interstitial granulomas	Associated vascular thrombotic lesions Emphysema Massive fibrosis	Polarized light examination: birefringent particles
*Bacterial Infections* Silico-tuberculosis	Epithelioid granulomas peripheral to silicotic nodules with central necrosis			
*Bacterial Infections* Nontuberculous atypical mycobacteria	Well-formed granulomas; less tendency to caseate than TB	Lung parenchyma (upper lobes)	Bronchiectasis: bronchocentric granulomas Necrosis with serpiginous borders and vasculitis (different from GPA)	Ziehl-Neelsen stain Fluorochrome but difficult to differentiate from *M. tuberculosis*
*Bacterial Infections* Hot-tub lung (*Mycobacterium avium intracellulare*)	Well-formed, nonnecrotizing, often coalescent granulomas	Bronchiolocentric and randomly distributed within airspaces Granulomas in the lumen of bronchioles Air-space granulomas		Organizing pneumonia Mild interstitial pneumonia
*Bacterial Infections* Melioidosis (tropical regions)	Granulomatous pattern: loose granulomas with necrosis Palisading histiocytes	Lung parenchyma	Necrotizing inflammation	Gram stain (*Burkholderia pseudomallei*: Gram-negative)
*Bacteria Infections* *Tropheryma whipplei*	Poorly formed granulomas composed of large clusters of histiocytes Foamy macrophages filled with bacteria or bacterial debris True granulomas in lymph nodes	Mediastinal lymph nodes Patchy, peribronchial and peribronchiolar granulomas Airway mucosa Perivascular Pleural		Gram-positive PAS-positive Grocott methenamine silver stain Thin sickle-shaped inclusions (*Tropheryma whipplei*)
*Fungal infections* *Cryptococcus* *Coccidioides* *Blastomyces* *Zygomycetes*	Nodular granulomas Necrotizing granulomas Giant cells containing yeasts	Lymph nodes Lung parenchyma Pleura	Coexisting areas of necrosis	Gram-positive PAS-positive Grocott methenamine silver stain Fontana-Masson stain
	Suppurative granulomas Granulomas with focal necrosis Granulomas with purulent exudate	Angioinvasive		
*Fungal infections* *Pneumocystis jirovecii*	Necrotizing granulomas (5–17%)	Lymph nodes: uncommon Lung parenchyma: collected cysts in subpleural areas	Lymphoplasmacytic interstitial infiltration	PAS Grocott methenamine silver stain
*Parasites* *Schistosoma mansoni*	Granulomatous vasculitis in association with eggs		Granulomatous vasculitis: arteritis	Observed eggs (measuring about or more than 100 mm)
*Parasites* *Paragonimus*	Granulomatous reaction associated with eggs	Adjacent to large bronchioles	Small cysts surrounding worms	Observed eggs (measuring less than 100 mm)
Sjögren’s syndrome	Small nonnecrotizing granulomas	Sparse and randomly distributed over the pulmonary interstitium	Peribronchial lymphoid hyperplasia Narrowing of the small airways Occasional bullae	
Granulomatous pneumonitis associated with Crohn’s disease	Crohn’s: scattered tiny nonnecrotizing granulomas	Crohn’s: rarely, randomly distributed	Crohn’s: related to drugs used to treat the disease	

##### 3.1.4.1. Pathological features of pulmonary sarcoidosis

Pulmonary sarcoidosis is defined as a systemic disease manifesting chiefly as granulomatous interstitial pneumonia. Consequently, the identification of characteristic noncaseating epithelioid granulomas is essential to the diagnosis.

In sarcoidosis, the epithelioid-cell granulomas are well-formed structures whose compact core is composed of macrophages and macrophage-derived cells (epithelioid and giant cells), closely associated with CD4+ T lymphocytes. The peripheral component contains CD8 lymphocytes, CD4+ FOXP3 + Treg cells, Th17 cells, B lymphocytes, and IgA-producing plasma cells (53–55) (Figure 5A). A feature that is common but not specific of sarcoidosis is the presence of cytoplasmic inclusions, chiefly within multinucleate giant cells. These inclusions may be Schaumann bodies (conchoidal bodies) made of iron and calcium salts (Figure 5B), crystalline bodies composed of calcium oxalate and carbonate, and/or asteroid bodies made of cholesterol (Table 2). Focal coagulative necrosis is occasionally seen in the center of the granulomas. Importantly, sarcoid granulomas are particularly florid and tend to coalesce. They are observed in 54–90% of **bronchial mucosal** biopsies, whose number strongly affects the diagnostic yield (56). The bronchi may be more severely affected, and classic endobronchial sarcoidosis is characterized by waxy, yellow, nonuniform, mucosal nodules 2 to 4 mm in diameter that are more profuse in the lobar and segmental bronchi and mimic a malignant mass (57, 58). A key feature of sarcoidosis is the presence in the **hilar and mediastinal lymph nodes** of variously sized, noncaseating, epithelioid granulomas (Figure 5A). At the early phase, small epithelioid-cell nodules develop in the cortex. Subsequently, well-demarcated granulomas are visible throughout the lymph node and may coalesce. At the late phase, fibrosis and hyalinization develop (Figure 5C) (53). Needle aspiration of intrathoracic lymph nodes under ultrasonographic guidance, either from the airways (transbronchial needle aspiration) or from the esophagus (transesophageal needle aspiration), is now the method of choice for mediastinal-node sampling in patients with presumed stage 1 or 2 sarcoidosis (59, 60). In the **lung**, collections of granulomas may produce macroscopically visible but small white nodules (micronodules) or large masses (macronodules), with relative sparing of the intervening lung. The nodules predominate along the lymphatics (collecting lymphatics in the pleural interstitium, interlobular septa, bronchovascular interstitium, and intralobular lymphatics). This topographic distribution is a very strong argument for pulmonary sarcoidosis. It suggests a critical role for lymphatics in nodule development and, consequently, supports the involvement of airborne particles in the pathogenesis of pulmonary sarcoidosis. In addition, given the presence of granulomas near the small airways, peribronchiolitis with bronchiole-lumen narrowing is common. Furthermore, the blood vessels are often involved in areas of granulomatous inflammation. Granulomatous vasculitis may affect all levels from the large elastic pulmonary arteries to the venules, although the lesions are more marked in the veins than in the arteries. Necrotizing sarcoid granulomatosis, first reported in 1973, is characterized by extensive noncaseating granulomatous inflammation with foci of parenchymal necrosis, combined with marked vasculitis affecting both the arteries and the veins (61). The granulomas may resolve without sequelae or leave fibrotic changes. Concentric fibrosis surrounding the granulomas is nearly always found. In addition, progressive pulmonary fibrosis with interstitial thickening by hyalinized fibrous tissue may be responsible for interstitial parenchymal fibrosis, which ultimately results in end-stage lung disease.

##### 3.1.4.2. Pathological features in the alternative diseases

Epithelioid granulomas develop in many infectious and non-infectious diseases (62–66). For example, the granulomas in chronic beryllium disease closely resemble sarcoid granulomas (Figure 5D). Inorganic agents (e.g., aluminum) can be identified by polarized light examination (Figures 5E,F) or special methods (67). The pathologist must consider all the lung diseases in which the organization of macrophages and macrophage-derived cells, with variable numbers of lymphocytes, resembles that seen in sarcoidosis. A granulomatous reaction *per se* is not specific of sarcoidosis, as shown in Table 2, and may be an immunological and inflammatory response to factors such as bacteria (e.g., *Mycobacterium tuberculosis* (Figures 5G,J) or nontuberculous mycobacteria). Controversial examples are hot-tub lung (68, 69) and Whipple’s disease (70) (Figures 5M,N). Fungal infections (e.g., *Histoplasma capsulatum, Cryptococcus, Pneumocystis*) may be misdiagnosed (Figures 5K,L) (71). Parasites (e.g., *Schistosoma mansoni*) may produce arterial granulomas responsible for pulmonary arterial hypertension (72). Exposure to organic airborne agents can cause hypersensitivity pneumonitis characterized by poorly formed granulomas that are often difficult to identify (65). In addition, granulomas develop in various systemic diseases including common variable immune deficiency, granulomatosis with polyangiitis, eosinophilic granulomatosis with polyangiitis, Sjögren’s syndrome, and Crohn’s disease. Figure 5O illustrates the various granuloma patterns, with the characteristics and extent of necrosis, and lists the methods for identifying causative agents (e.g., special stains and polarized light examination). These distinctive features are summarized in Table 2.

##### 3.1.4.3. Bronchoalveolar lavage

BAL is a safe and minimally invasive procedure that has been widely performed for nearly 40 years (73). Its relevance for diagnosing diffuse interstitial lung disease remains debated (65, 74). However, in patients with sarcoidosis, BAL allows the demonstration of CD4+ T-cell lymphocytic alveolitis (75). Although this finding is not specific, once infections have been ruled out, a lymphocyte count above 25% strongly suggests sarcoidosis, hypersensitivity pneumonitis, or drug toxicity (65, 76, 77). In sarcoidosis, moderate lavage-fluid lymphocytosis (20%–50%) is found in 80% of cases, and the CD4/CD8 T-cell ratio is higher than 3.5 in 50% of cases. The CD4/CD8 T-cell ratio is of controversial diagnostic relevance and, in practice, should be considered informative only when above 3.5, when its specificity is 93%–96% but its sensitivity only 53%–59% (78). Studies that used integrated differential analyses of lavage-fluid cells achieved either by a computer program based on a logistic model (79) or by Bayesian analysis (80) suggested that low percentages of lymphocytes, neutrophils, and eosinophils combined with a high CD4/CD8 T-cell ratio may raise the likelihood of sarcoidosis to more than 85% (80). BAL, although perhaps not decisive for the diagnosis, has a major role to play in research on sarcoidosis. Thus, recent data obtained using this investigation have generated new hypotheses about the possible role in sarcoidosis for B cells (81) and for causal agents such as *Aspergillus* antigens (82).

#### 3.1.5. Microbiology

##### 3.1.5.1. Microbiology of mycobacteria

The prompt and accurate diagnosis of pulmonary tuberculosis is essential. To establish a definite diagnosis of pulmonary tuberculosis, mycobacteria of the *M. tuberculosis* complex must be identified in pathological samples such as respiratory secretions (e.g., sputum), lymphadenopathy, other infected tissues, or pleural fluid. The microbiology laboratory must perform microscopic examinations and cultures according to standardized procedures (83, 84). The diagnosis of tuberculosis relies on the interpretation of findings from multiple tests including the microscopic examination of stained pathological samples, cultures, and genome-amplification tests designed to identify *M. tuberculosis* complex organisms.

The microscopic examination of smears takes less than 1 hour. Two methods are used routinely: (i) the auramine O fluorescent stain, usually chosen for screening, with a reading at X25 or X40 magnification and (ii) the Ziehl-Neelsen stain, used to confirm a positive auramine O test, with a reading at X100 magnification. Culturing is the reference test, since it identifies a bacterial strain belonging to the *M. tuberculosis* complex, but at least 10 to 30 days are required.

*M. tuberculosis* complex detection based on polymerase-chain-reaction (PCR) gene amplification of samples (PCR-TB test) is a routine investigation recommended for diagnosing tuberculosis (85, 86). When applied to samples positive by microscopy, this test can identify *M. tuberculosis* complex organisms within a few hours, thereby allowing appropriate patient management before the culture results are available (87–89).

The microscopic examination of stained smears has two main limitations: (i) it does not reliably distinguish between *M. tuberculosis* complex and nontuberculous mycobacteria and (ii) its sensitivity is lower compared to cultures, as the results are positive only with loads above 5,000 to 10,000 bacilli/mL of sample compared to 1 bacillus/mL, in theory, for cultures. Smear examination can rapidly detect those patients with the highest bacillus loads and account for about 50–60% of patients with culture-positive pulmonary tuberculosis (89–91). Sensitivity increases with the number of samples examined with a sensitivity of 74%, 86%, and 92% with one, two, and three samples, respectively (92).

Culturing is the reference standard test for diagnosing tuberculosis. The samples are first decontaminated and centrifuged to kill nontuberculous bacteria (pyogenic and commensal bacteria), which grow faster than do mycobacteria and can contaminate cultures (93). The sample is then inoculated either onto solid Löwenstein-Jensen medium, which is incubated at 35°C–37°C and produces visible colonies within 21 to 28 days, or into liquid medium, which can produce colonies within 10 to 21 days. A microscopic examination is then performed to confirm the presence of acid-alcohol resistant bacteria. With solid medium, the results are expressed quantitatively as the number of colonies per tube. With liquid medium, the time to a positive detection signal correlates with the amount of bacilli, thus providing a semi-quantitative estimate. The culture is reported as negative after 42 days with liquid medium and 60 days with solid medium. The identification of mycobacteria recovered by culturing was classically based on several cultural and biochemical features. This method is being increasingly replaced by molecular techniques (RNA/DNA hybridization, amplification of specific gene regions or insertion sequences) or by an immunochromatographic test that detects the MPT64 antigen (83).

An alternative method relies on matrix-assisted laser desorption ionization-time of flight mass spectrometry (MALDI-TOF MS), which provides a characteristic mass spectral fingerprint of whole inactivated mycobacterial cells. This method is accurate for identifying bacteria. Its simplicity, speed, and affordability make it suitable as a routine technique for identifying mycobacteria (94, 95).

Many tests marketed since the 1990s use gene-amplification techniques (PCR-TB tests) to identify mycobacteria of the *M. tuberculosis* complex within a few hours (standard or real-time PCR, ligase chain reaction, transcription-mediated amplification, nucleic-acid sequence-based amplification, isothermal amplification) (87, 96–99). Automated PCR tests (notably the Xpert® MTB/RIF assay) can detect *M. tuberculosis* complex and rifampicin resistance simultaneously, generally within 2 hours. Overall, sensitivity of these tests is about 80%–90% with culture-positive respiratory samples but only 60%–70% with smear-negative samples (85). Specificity is 98%–99% (85, 86, 100).

With extrapulmonary samples, the larger the volume the greater the likelihood of identifying the bacteria. For example, for possibly tuberculous lymph nodes, needle aspiration alone has a limited diagnostic yield, except in countries where tuberculosis is uncommon or when a PCR test is performed on the aspirate (101). However, PCR requires special equipment, reagents, and technician time that may not be available in low-income countries.

##### 3.1.5.2. Microbiology of nonmycobacterial microorganisms

Histoplasmosis is diagnosed either by identification of the organism upon microscopic examination and culturing of sputum or tissue samples or by PCR testing of whole blood. *Histoplasma capsulatum* is a dimorphic fungus. Urine can be tested for *Histoplasma* antigen.

Several bacteria other than mycobacteria may cause symptoms suggestive of sarcoidosis and should be sought using standard bacteriological tests before considering tests for organisms that are more challenging to identify. Most of these bacteria are easily recovered by culturing then identified by routinely available MALDI-TOF mass spectrometry. Unlike *M. tuberculosis*, these bacteria do not require any special precautions in the laboratory. If the standard cultures are negative, PCR tests for the 16S rRNA gene can be performed as they may, in some cases, identify the causative bacterial agent.

Syphilis and leprosy do not typically damage the lung parenchyma. A single case of syphilitic granuloma in the lung parenchyma has been reported (102). The diagnosis relies mainly on indirect methods, i.e., serology or PCR testing, which are performed only in selected cases.

To look for viruses such as the human immunodeficiency virus, varicella-zoster virus, and cytomegalovirus, specific tests must be ordered. The diagnosis is usually achieved by specific PCR or serological tests, which are selected based on the clinical presentation, country of residence, and local epidemiology.

Metagenomic next-generation sequencing may, in the future, prove a useful and rapid tool for detecting bacteria, fungi, or viruses in granulomatous tissue. Advantages of this technique include the simultaneous identification of multiple pathogens, detection of microorganisms that are present in small amounts or difficult to grow, and feasibility on formalin-fixed and paraffin-embedded tissue.

#### 3.1.6. Other investigations

Increased serum angiotensin converting enzyme level and abnormal calcium and vitamin D metabolism are often observed in sarcoidosis. However, both abnormalities are neither sensitive nor specific. They can be abnormal in several other granulomatous conditions particularly tuberculosis and CBD and even in non-granulomatous conditions like lymphomas.

^18F^Fluodeoxyglucose (FDG) positron emission tomography (PET) CT (PET/CT) is the most sensitive test to assess activity in involved organs in sarcoidosis and no study has been done to assess its usefulness for diagnosis. There are not dedicated studies comparing PET/CT in sarcoidosis and other granulomatous or non-granulomatous diseases like for example lymphomas. Moreover, its use has not been recommended at diagnosis except in rare cases where a cardiac sarcoidosis is suspected (1).

### 3.2. More on alternative diseases: when can they mimic sarcoidosis and which tools can establish the differential diagnosis?

#### 3.2.1. Infections

In Western European countries, Africa, and Asia, tuberculosis is the leading differential diagnosis of sarcoidosis. In North America, in contrast, nontuberculous mycobacterial infections and histoplasmosis may be more common than tuberculosis.

##### 3.2.1.1. Tuberculosis

Tuberculosis is the main differential diagnosis in most countries. The importance of assessing the epidemiological setting is highlighted above (section 3.1.1.) The prompt and accurate diagnosis of pulmonary tuberculosis is essential.

###### 3.2.1.1.1. When is the differential diagnosis most difficult?

Sarcoidosis is particularly difficult to diagnose in patients with rare and atypical radiological presentations such as unilateral hilar or mediastinal lymphadenopathy, necrotic lymphadenopathy, predominantly bronchiolar micronodular infiltration or miliary, lung cavitations, or pleural effusion. These presentations should suggest tuberculosis, particularly in endemic regions (section 3.1.1.). The absence of caseating granulomas does not rule out tuberculosis (103). In all patients, including those with imaging-study findings typical for sarcoidosis, mycobacteria should be sought in biopsy specimens and, if performed, by BAL.

###### 3.2.1.1.2. How to definitively rule out tuberculosis

Tuberculosis is easily ruled out when the following criteria are combined: country with a low incidence of tuberculosis, no contact with tuberculosis patients, radiological findings typical for sarcoidosis (section 3.1.3.), absence of clinical or imaging-study findings suggestive of tuberculosis, and negative microbiological tests on respiratory samples (sputum, BAL fluid) or tissue specimens (obtained by endobronchial ultrasound or other methods and examined using specific stains and microbiological techniques, section 3.1.5.1.).

However, in countries where tuberculosis is endemic (e.g., India), tuberculosis is more difficult to definitively rule out at presentation, as shown by a recent study of the Sarcoidosis Diagnostic Scores (7). Sarcoidosis often produces misleading constitutional symptoms such as fever, weight loss, and fatigue (104). In a study of patients with sarcoidosis in India, the lymph nodes appeared necrotic or nonhomogeneous by chest CT in 5.9% of cases, and necrotizing granulomas were found in tissue samples in 13.5% of cases (104). Stronger arguments for sarcoidosis are tuberculin anergy (71.9% of cases), typical isolated bilateral lymphadenopathy, and absence of necrosis by CT and/or histopathology. In tuberculosis-endemic countries, the proportion of presentations mimicking sarcoidosis is, as everywhere else, low but the high absolute number of tuberculosis cases increases the risk of confusion with sarcoidosis, particularly as microbiological studies for *M. tuberculosis*, including PCR tests, can produce false-negative results (105). Moreover, the tuberculin skin test and interferon-γ release assay can be negative despite confirmed tuberculosis, notably in elderly patients or when the peripheral lymphocyte count is low (106–108). In doubtful cases, the initiation of tuberculosis treatment is often wise, as the diagnosis of sarcoidosis may be confirmed only by the clinical course during tuberculosis treatment, which usually differs between tuberculosis and sarcoidosis.

Interestingly, in one study, the risk of developing sarcoidosis was significantly increased in patients with tuberculosis, particularly involving extrapulmonary sites (109). Moreover, the two diseases may co-exist at presentation. In this situation, only the accumulation of evidence over time can clarify the diagnosis.

##### 3.2.1.2. Nontuberculous mycobacteria

The clinical presentation and imaging-study findings closely resemble those seen in tuberculosis. Several specific features that vary across mycobacterial species deserve note. Fibrocavitary lesions in the lung apices are more common with *Mycobacterium kansasii* and *Mycobacterium malmoense*, whereas bronchiolar micronodules with a tree-in-bud appearance and bronchiectasis more often indicate *Mycobacterium avium* complex (MAC) infection. Isolated or multiple nodules can be found in *M. avium* complex and *Mycobacterium xenopi* infections.

To rule out nontuberculous mycobacterial infections, microbiological studies should be performed, as they usually allow the isolation and identification of mycobacteria. However, the diagnosis may be difficult in patients with multiple pulmonary nodules by chest CT, since investigations for mycobacteria in respiratory secretions or BAL fluid may be falsely negative. In this situation, the only definitive test is a surgical biopsy examined using specific microbiological techniques (section 3.1.5.1.).

##### 3.2.1.3. Histoplasmosis

Histoplasmosis may be a differential diagnosis of sarcoidosis in several circumstances.

###### 3.2.1.3.1. When is the differential diagnosis most difficult?

When the presentation is acute, imaging studies may be misleading if they show both lymphadenopathy, which may be bilateral, and diffuse lung micronodules. However, the clinical presentation more closely resembles that of community-acquired pneumonia, and the epidemiological setting is suggestive (section 3.1.1.).

In everyday practice, the differential diagnosis with sarcoidosis is most difficult in patients with (i) multiple chronic pulmonary nodules or (ii) pulmonary hypertension revealing previously unrecognized late-stage mediastinal fibrosis.

###### 3.2.1.3.2. How to definitively rule out histoplasmosis

Histoplasmosis occurs in specific epidemiological settings (section 3.1.1.) The diagnosis relies on the identification of the causal organism by microscopic examination of sputum or tissue and by culturing or PCR testing of whole blood.

##### 3.2.1.4. Other infections

Other bacteria that may cause symptoms and chest-imaging findings suggestive of pulmonary sarcoidosis are very rare. Q fever is usually diagnosed indirectly, by serological or PCR testing. Whipple’s disease is an infection by the Gram-positive bacillus *Tropheryma whipplei*. Patients usually present with weight loss (93%), diarrhea (81%), and arthralgia (73%). Pulmonary involvement is extremely rare and produces diffuse pulmonary ground-glass opacities predominating in the lower subpleural areas, pleural involvement, and noncaseating pulmonary granulomas (70, 72, 110, 111). The diagnosis relies on the identification of *T. whipplei* in various samples, including BAL fluid, using quantitative PCR (111). The BAL fluid shows neutrophilia and eosinophilia, as opposed to lymphocytosis, and stains with periodic acid-Schiff. As indicated above (section 3.1.5.2.), syphilis is an exceedingly rare cause of lung granulomatosis.

Tests for viruses such as the varicella-zoster virus and cytomegalovirus should be selected based on the clinical history and epidemiology. Persistent diffuse lung micronodules can be found long after typical adulthood varicella. Although COVID-19 does not appear to cause granulomas, possibly coincidental sarcoidosis-like disease diagnosed shortly after COVID-19 onset has been reported in three patients (112).

Several fungal infections other than histoplasmosis can cause granulomatous lung diseases that usually mimic tuberculosis. These infections occur chiefly in immunocompromised patients, but rare cases have been reported in immunocompetent individuals. They include cryptococcosis, coccidioidomycosis, and mucormycosis (113–115).

Parasitic infections cause pulmonary granulomatous diseases only very rarely and only in endemic countries. Leishmaniosis and paragonimiasis are examples, as well as schistosomiasis due to *S. mansoni*, which can present as pulmonary arterial hypertension with liver fibrosis and portal hypertension (72, 116, 117).

#### 3.2.2. Occupational and environmental diseases

##### 3.2.2.1. Chronic beryllium disease

Chronic beryllium disease can develop in individuals who are exposed to beryllium, usually at the workplace, and develop sensitization to this metal (118, 119). The features of the pulmonary granulomatous disease caused by beryllium closely match those of pulmonary sarcoidosis.

###### 3.2.2.1.1. When is the differential diagnosis most difficult?

The circumstances of the diagnosis vary widely. Chronic beryllium disease is readily diagnosed when the manifestations are detected during routine workplace monitoring of workers exposed to beryllium. However, individuals may be unaware of the exposure. Unless a systematic and detailed occupational history is obtained, sarcoidosis is often erroneously diagnosed at first, given the similarities in radiological, serum angiotensin-converting enzyme assay, BAL, and histopathology findings (120, 121). Moreover, the tuberculin test is often negative in chronic beryllium disease. Interestingly, gene expression patterns in peripheral-blood mononuclear cells were not different between patients with chronic beryllium disease and sarcoidosis (122). The only reported differences are rare extrapulmonary manifestations and rare voluminous hilar and mediastinal lymphadenopathy in chronic beryllium disease (118). In one study, among 84 patients with potential beryllium exposure and suspected or diagnosed sarcoidosis, 34 were diagnosed as having chronic beryllium disease instead, based on a positive beryllium lymphocyte proliferation test (120). Of these 34 patients, 28 had first been diagnosed with sarcoidosis, the median time between the two diagnoses being 3 [0.25–18] years.

###### 3.2.2.1.2. How to definitively rule out chronic beryllium disease

Differentiating chronic beryllium disease from sarcoidosis relies chiefly on two investigations: first, a systematic and detailed occupational history including questions about possible occupational beryllium exposure should be obtained in every patient with suspected pulmonary sarcoidosis and, second, a beryllium hypersensitivity test is mandatory in all patients with a history of beryllium exposure, even in the distant past.

Occupational beryllium exposure may occur during primary beryllium and beryllium-alloy production; in dental laboratories; in nuclear power plants; during nuclear weapon manufacturing; and during aerospace or ceramic manufacturing (119). Beryllium exposure has also been reported in security guards, accountants, exposed workers’ spouses, and people living near beryllium-production facilities. Exposure may be demonstrated unexpectedly, as shown recently in workers who inhaled concrete dusts containing high beryllium concentrations (123).

Beryllium hypersensitivity can be demonstrated by performing a beryllium lymphocyte proliferation test. The result is abnormal when the lymphocyte count is greater than 15%. Two or more abnormal results on blood, one abnormal and one borderline result on blood, or one positive result on BAL fluid confirms beryllium hypersensitivity (119). In clinical practice, the absence of an occupational history of beryllium exposure combined with two negative beryllium lymphocyte proliferation tests on blood rule out chronic beryllium disease.

HLA-DPB1 alleles encoding a glutamic acid residue at position 69 of the β-chain (Glu69) are associated with an increased risk of both beryllium hypersensitivity and chronic beryllium disease, with odds ratios greater than 10 (118). However, the Glu69 variant can be found in healthy individuals and is absent in 25% of patients with chronic beryllium disease. Thus, HLA-DPB1 testing has no role as a diagnostic investigation for chronic beryllium disease.

##### 3.2.2.2. Non-beryllium-metal-induced granulomatosis

Positive lymphocyte transformation tests to metals other than beryllium have been reported in patients with sarcoidosis-like presentations and workplace exposures to metals (e.g., during jewelry manufacturing or welding). The metals/minerals were aluminum, nickel, titanium, chromium, palladium, silica, mercury, and zirconium. Thus, some patients given a diagnosis of sarcoidosis may have metal-induced sarcoidosis-like granulomatous disease (124, 125).

##### 3.2.2.3. Hypersensitivity pneumonitis

Hypersensitivity pneumonitis, notably in its nonfibrotic form, is caused by exposure to airborne environmental or occupational antigens and can share features with sarcoidosis. However, differentiation from sarcoidosis is generally easy, as shown by a study of the Sarcoidosis Diagnostic Scores (7). The onset may be acute, subacute, or insidious, and recurrent episodes may develop. Crackles are common and squawks may be heard. Although many patients have constitutional symptoms including weight loss, the disease is limited to the lungs, without extrapulmonary organ involvement. At least 40% of patients have either exposure to or serum IgG against an environmental or occupational inciting agent (e.g., avian antigens, bacteria, mycobacteria, fungi, isocyanates). The imaging findings typically differ from those in sarcoidosis (section 3.1.3.) (76, 126). Although BAL fluid lymphocytosis occurs in both hypersensitivity pneumonitis and sarcoidosis, a relative lymphocyte count above 50% has been reported in half the patients with the former versus almost none with the latter (80). In BAL fluid, the total cell count, percentages of neutrophils and eosinophils, and percentage of mast cells — this last having the greatest discriminating power — tend to be higher in hypersensitivity pneumonitis than in sarcoidosis, whereas the CD4/CD8 T-cell ratio tends to be above normal in sarcoidosis and below normal in hypersensitivity pneumonitis (80, 127). Combining the history with blood-test, imaging-study, and BAL findings usually suffices to differentiate hypersensitivity pneumonitis from sarcoidosis. The granulomas found in transbronchial lung-biopsy specimens in hypersensitivity pneumonitis are typically small and poorly formed and tend to predominate in the peribronchiolar interstitium (section 3.1.3.). This finding combined with those of the above-listed investigations is highly discriminating.

##### 3.2.2.4. Hot-tub lung

Hot-tub lung is an infrequent interstitial lung disease secondary to the inhalation of aerosolized hot-tub water containing nontuberculous mycobacteria (*M. avium or Mycobacterium phocaicum*) (128). Chest CT findings may closely resemble those in hypersensitivity pneumonitis, with diffuse ground-glass opacities and mosaic attenuation. However, the lung biopsy shows different features consisting in nonnecrotizing and mostly well-formed granulomas in a bronchocentric distribution. Differentiation from sarcoidosis is easy even in the absence of a lung biopsy, based on (i) use of a hot tub, (ii) the very different CT images, and (iii) the recovery of nontuberculous mycobacteria from respiratory specimens.

##### 3.2.2.5. Granulomatous talcosis

Pulmonary granulomatous talcosis can produce radiological abnormalities resembling those seen in sarcoidosis (section 3.1.3.) (39). A history of inhaled or intravenous drug abuse suggests the diagnosis. Confirmation is provided by lung biopsy, which shows small diffuse granulomas at the level of the terminal or respiratory bronchioles, with birefringent bodies within giant cells by polarized light microscopy (129). These lesions are more similar to foreign-body granulomas than to sarcoid granulomas.

#### 3.2.3. Drugs

A recent WHO pharmacovigilance review reported 2,425 cases of drug-induced sarcoidosis and strong associations with TNF-a antagonists, interferon, pegylated interferon, and immune checkpoint inhibitors (19, 130, 131).

##### 3.2.3.1. TNF-a antagonist-induced granulomatous diseases

Despite data supporting the use of TNF-a antagonists to treat sarcoidosis, these drugs have been reported to induce sarcoid-like reactions (18, 132). Etanercept was most often involved, but cases were also seen with adalimumab, infliximab, golimumab, and biosimilars. The reason for TNF-a antagonist therapy was usually rheumatoid arthritis, although some patients had ankylosing arthritis, Crohn’s disease, or even sarcoidosis (133–136). TNF-a antagonist-induced granulomatous disease is rare; thus, only four (0.57%) cases were identified among 697 patients with rheumatoid arthritis (133). The mean duration of TNF-a antagonist exposure has varied widely, from 7 to 123 months in rheumatoid arthritis and 1 to 180 months in Crohn’s disease. Diffuse lung infiltration with or without hilar lymphadenopathy was common. Peripheral lymphadenopathy, uveitis, and involvement of the spleen, muscle, parotid glands, and spinal cord have also been reported. Laboratory test abnormalities included hypercalcemia, which was severe in some patients, and elevated serum angiotensin-converting enzyme levels. Diagnostic challenges arise due to the low incidence of TNF-a antagonist-induced granulomatosis, wide variability in exposure duration at symptom onset, and possible presence of symptoms due to the underlying disease (e.g., rheumatoid arthritis or Crohn’s disease) (137, 138). The prompt fading of the manifestations after withdrawal of the suspected drug strongly supports TNF-a antagonist-induced granulomatous disease. In patients with sarcoidosis, worsening of the disease during TNF-a antagonist therapy is difficult to interpret, particularly given the paucity of relevant information in the literature. A relapse after an initial response to TNF-a antagonist therapy may indicate either drug-induced granulomatous disease or the development of antidrug antibodies, as reported with infliximab or adalimumab in patients with rheumatoid arthritis (139, 140). Antidrug antibody assays are very useful in this situation.

##### 3.2.3.2. Sarcoid-like reaction to anticancer drugs

Some of the most recent anticancer drugs including immunotherapeutic agents and targeted therapies have been incriminated in the development of granulomas. The recent WHO pharmacovigilance report recognizes PD-1, CTLA4 (103 cases), and BRAF and MEK inhibitors (37 cases) as causes of sarcoid-like granulomatosis (130).

###### 3.2.3.2.1. Immune checkpoint inhibitors

Pulmonary granulomatosis was first described with CTLA-4 inhibitors then with PD-1 and PD-L1 inhibitors. In several patients, the CTLA-4 inhibitors ipilimumab and tremelimumab seem to have caused cutaneous, lymph-node, and lung involvement with noncaseating granulomas, as well as lymphocytic alveolitis (141–144). Time to symptom onset was usually three to 6 months. The PD-1 inhibitor nivolumab has been associated with granulomatosis of the lungs and of other organs including the eyes (145). This appears to be a class effect, as granuloma formation has been reported with the PD1 inhibitors pembrolizumab (146) and nivolumab (147) and with the PD-L1 inhibitors atezolizumab (148), durvalumab (149), and avelumab (150). The time to onset in patients given both CTLA-4 and PD-1 inhibitor therapy may be shorter, as with all toxicities from immunotherapies (151). Retrospective data suggest that a sarcoid-like reaction may be associated with a better prognosis. Of 434 retrospectively reviewed patients with adverse effects of checkpoint inhibitor therapy, 28 had sarcoid-like reactions, which were asymptomatic in half the cases and often manifested only as mild dyspnea or a cough with no serious lung impairments in the other cases. The most common primary cancer was melanoma and most patients with taking both CTLA-4 and PD-1 inhibitors. Compared to the 406 patients with other adverse events, those with sarcoid-like reactions had better overall survival (hazard ratio, 0.232; 95% confidence interval, 0.086–0.630; *p* = 0.002) in this retrospective study (152).

Importantly, patients with sarcoid-like reactions are at risk of being misdiagnosed either with cancer progression in the absence of histological studies or with an infection precipitated by the treatment-induced immunosuppression (combined chemotherapy and immunotherapy). Sarcoid-like reactions can mimic cancer progression. Of 45 patients given adjuvant combined nivolumab-ipilimumab therapy for melanoma, 10 (22%) were diagnosed with sarcoid-like reactions manifesting most often as hilar and mediastinal lymphadenopathy and in some patients as skin and bone involvement (153). An unresolved issue is whether the type of primary cancer and/or disease stage affect the incidence of sarcoid-like reactions (153). Findings that strongly support a need for histological studies include atypical changes in the radiological abnormalities, a dissociated radiological response, and a marked discrepancy between the radiological and clinical signs.

###### 3.2.3.2.2. Targeted therapies

Other anticancer treatments that can induce sarcoid-like reactions include BRAF and MEK inhibitors. Cases have been reported with the BRAF inhibitor vemurafenib used alone (154) or with the BRAF inhibitor dabrafenib combined with a MEK inhibitor (trametinib or cobimetinib) (155, 156). Bilateral hilar lymphadenopathy, uveitis, and cutaneous involvement have been reported (154). These manifestations were usually not severe, and the cutaneous and ophthalmological abnormalities resolved promptly with topical treatment despite continuation of the anticancer drugs. In one patient, however, acute kidney injury required discontinuation of the targeted treatment and the administration of corticosteroid therapy (155).

###### 3.2.3.2.3. Intravesical BCG therapy for bladder cancer

Intravesical BCG therapy for bladder cancer can cause systemic granulomatosis (157, 158). Granulomatous pneumonitis, sometimes with systemic involvement, has been reported in 0.7%–0.9% of patients (159, 160). The pathogenesis of BCG-related granulomatous pneumonitis is debated but may involve a hypersensitivity reaction to disseminated BCG (158).

The chest radiograph may be normal or show a miliary pattern mimicking disseminated tuberculosis, a fungal infection, or hematogenous metastases (158).

##### 3.2.3.3. Interferon-induced pulmonary granulomatous disease

New treatments for hepatitis have largely superseded interferon-a, except for patients with hepatitis delta. Interferon-induced pulmonary granulomatous disease has therefore become rare.

##### 3.2.3.4. Highly active antiretroviral therapy (HAART) for HIV infection

A sarcoidosis-like disease can develop in HIV-positive patients who have responded to antiretroviral therapy by a rise in CD4 T-cell counts and a fall in viral loads. In 10 retrospectively identified HIV-positive patients with a newly diagnosed, sarcoidosis-like disease, the chest CT findings resembled those produced by sarcoidosis in HIV-negative patients: they consisted of lymphadenopathy, nodules, thickened interlobular septa, focal consolidation, reticular opacities, ground-glass opacities, and cyst-like or other cavities (161). In HIV-positive patients receiving HAART, sarcoidosis-like manifestations may indicate true sarcoidosis related to restoration of the immune system.

##### 3.2.3.5. Other drug-induced pulmonary granulomatous diseases

The monoclonal antibodies rituximab, omalizumab, ustekinumab, vedolizumab, and natalizumab are suspected causes of pulmonary granulomatosis (130, 162, 163). Mesalamine has been suggested to induce pulmonary granuloma formation when used to treat Crohn’s disease.

#### 3.2.4. Immune deficiencies

##### 3.2.4.1. Pulmonary granulomatosis-associated common variable immunodeficiency

This disease often results in recurrent bacterial respiratory infections (65% of cases), bronchiectasis, and autoimmune manifestations (40% of cases) and may co-exist with lymphoproliferative disorders (21). However, the disease may remain asymptomatic until sarcoid-like abnormalities develop. Findings may include mediastinal lymphadenopathy and bronchial or parenchymal granulomas (21). The pulmonary granulomatous lesions may be isolated or co-exist with organizing pneumonia, lymphoid interstitial pneumonitis, follicular bronchiolitis, or lymphoid hyperplasia. The term “granulomatous-lymphocytic interstitial lung disease” has been used to designate this condition but remains controversial (21, 164). Serum protein electrophoresis, which must be part of the diagnostic workup for sarcoidosis, shows hypogammaglobulinemia, thereby establishing the diagnosis (1). Importantly, the chest CT findings differ substantially from those seen in sarcoidosis (section 3.1.3.).

##### 3.2.4.2. Pulmonary manifestations in adults with chronic granulomatous disease

Chronic granulomatous disease is a rare inherited primary immunodeficiency caused by a mutation in the NADPH oxidase gene. The respiratory manifestations are major complications. The diagnosis is usually made in early childhood upon the evaluation of recurrent infections. The pulmonary infections are often chronic and may be asymptomatic, notably when caused by *Aspergillus fumigatus*. A biopsy of persistent pulmonary nodules or consolidations is often required and frequently shows noncaseating granulomas. Radiological presentations mimicking sarcoidosis are very rare (165, 166). The repeated infections starting at a very young age combined with the radiological presentation usually make sarcoidosis very improbable.

#### 3.2.5. Genetic diseases

Blau syndrome is an autosomal dominant disorder due to *NOD2* mutations, of which over 15 have been identified (167, 168). Familial cases are present in 40% of patients. The onset is usually at 3–4 years of age (15). Most patients have the typical triad of skin involvement, symmetrical polyarthritis, and uveitis. Lung involvement has been reported in a single patient, who had ground-glass opacities in the middle and lower lobes (169). The diagnosis is based on the presentation and results of genetic testing for *NOD2* mutations. Thus, Blau syndrome is easily differentiated from sarcoidosis (170).

#### 3.2.6. Vasculitides and autoimmune diseases

##### 3.2.6.1. Granulomatosis with polyangiitis

This disease typically manifests as necrotizing granulomatous inflammation of the ears, nose, and upper and lower respiratory tracts and as necrotizing vasculitis involving the small- to medium-sized vessels, often with glomerulonephritis (171).

The differential diagnosis with sarcoidosis is rarely difficult unless the lung is the only site involved, with condensations or noncavitated nodular lesions. However, differences with sarcoidosis include the absence of co-existing perilymphatic micronodular lesions and of lymphadenopathy (46). A positive assay for antineutrophil cytoplasmic antibodies directed to proteinase 3 (PR3-ANCA) is highly specific but only 60% sensitive. In doubtful cases, a lung biopsy, performed surgically to ensure the collection of sufficient material, may be indicated. The typical histological pattern is a triad of vasculitis, necrosis, and granulomatous inflammation, which may co-exist with organizing pneumonia or alveolar hemorrhage.

##### 3.2.6.2. Eosinophilic granulomatosis with polyangiitis

Lung granulomas can develop in eosinophilic granulomatosis with polyangiitis, producing a radiological presentation very similar to that seen in granulomatosis with polyangiitis. However, differentiation with sarcoidosis is readily achieved based on the history of asthma (often severe), marked blood eosinophilia, serum C-reactive protein elevation, and positive assays for perinuclear antineutrophil cytoplasmic antibodies.

##### 3.2.6.3. Necrotizing sarcoid granulomatosis

“Necrotizing sarcoid granulomatosis” is still a provisional diagnostic term, given the uncertainty about whether it represents necrotizing angiitis with a sarcoid reaction or true sarcoidosis with a distinctive pathological pattern (172). The epidemiology and presentation are very similar to those of sarcoidosis. However, thoracic lymphadenopathy occurs in only 33% of patients. The only clear difference lies in the pathological findings. However, a possible source of bias is that the lung specimens are often obtained surgically and are therefore larger than for sarcoidosis. The prevailing view at present is that necrotizing sarcoid granulomatosis is a form of sarcoidosis (172).

##### 3.2.6.4. Interstitial lung disease associated with Sjögren’s syndrome

Interstitial lung disease, sometimes with granulomatous pulmonary lesions, may develop in patients with sicca due to Sjögren’s syndrome. Very infrequently, this condition may require differentiation from sarcoidosis with pulmonary fibrosis producing linear opacities extending from the hilum to the subpleural area (173). However, the pathological features of granulomatous lesions in Sjögren’s syndrome differ markedly from those of sarcoid granulomas (section 3.1.4.).

#### 3.2.7. Crohn’s disease

Noninfectious pulmonary involvement has rarely been studied in patients with Crohn’s disease. The first diagnosis to consider is an adverse drug reaction, for instance to mesalamine or a TNF-a antagonist (174). In practice, Crohn’s disease is never *per se* the cause of pulmonary granulomatosis. At the trachea, in contrast, macroscopic abnormalities can be visible by endoscopy, a finding never observed in sarcoidosis (175). Crohn’s disease and sarcoidosis may co-exist (176).

#### 3.2.8. Granulomatosis associated with lymphoma and solid malignancies

Non-caseating granulomas have been reported in 0.7–13% of patients with malignancies (177) including lymphomas; testicular, breast, lung, and head-and-neck cancer; and melanoma (177, 178). Sarcoidosis-like granulomas were identified in 14% of patients with Hodgkin’s lymphoma and 7% with non-Hodgkin’s lymphoma (179, 180). The granulomas may develop within the tumor itself or in the regional draining lymph nodes (e.g., mediastinal nodes in lung cancer). Distant lymph nodes may be involved, and lung nodules may develop remotely from primary skin melanoma (178, 179). The granulomas may be identified only many years after the diagnosis of cancer. The main diagnostic challenge is differentiation from metastases. A granulomatous reaction to cancer is readily distinguished from sarcoidosis when it is present only in the neighborhood of the malignancy. Investigations for granulomas at other sites must therefore be performed. Sometimes, a typical sarcoidosis can be evidenced upon review of earlier investigations. In challenging cases, only the collection of further data during follow-up can establish the diagnosis.

The risk of lymphoma is 2-fold to 11-fold higher in patients with vs. without sarcoidosis (179, 181). Thus, manifestations that develop during the course of sarcoidosis may be due to lymphoma. On the other hand, clinical events in patients with lymphoma may be due to another disease. One study identified 14 new and 25 previously reported patients in whom sarcoidosis developed after a diagnosis of Hodgkin’s or non-Hodgkin’s lymphoma (182).

Lymphomatoid granulomatosis is a rare B-cell lymphoproliferative disease associated with Epstein–Barr virus infection. Multiple lung nodules predominating in the middle to lower lung fields may develop. The diagnosis is confirmed by the identification of atypical B cells positive for Epstein–Barr virus-encoded small non-polyadenylated RNA 1 and 2 (EBER). These cells may be sparse and associated with nonnecrotizing granulomatous inflammation (183).

## 4. Conclusion

Alternative diagnoses must be ruled out before a diagnosis of sarcoidosis can be given. To this end, a rigorous diagnostic strategy must be applied. First, epidemiological factors must be clarified, including local infectious diseases, regions of travel, occupational and environmental exposures, drug abuse, exposure to medications, and family history. A detailed medical history and thorough physical examination are also crucial, as they may, for instance, suggest an immunodeficiency or identify extrapulmonary abnormalities that may be characteristic of specific diseases. Chest CT is very helpful, particularly when read by a highly experienced radiologist who can characterize the findings as typical or atypical for sarcoidosis and for the alternative diagnoses. All other investigations are guided by this information. A study reported nearly three decades ago used Bayes’ theorem to diagnose chronic interstitial lung diseases based only on clinical and radiological findings (184). High confidence (greater than 95% probability) in a final correct diagnosis of sarcoidosis was obtained in 80% and 78% of patients in the training and validation sets, respectively. The improvements in CT acquisition and interpretation achieved since this study was done would probably result in even better performance. Another important message is the need to obtain typical granulomas for confirming diagnosis in most of the patients to avoid misdiagnosis with some alternative diseases shown to have a confusing presentation, particularly lymphoproliferative disorders. Eventually, using scores like the Sarcoidosis Diagnosis Scores Clinical and Biopsy can be very helpful to assess sarcoidosis diagnosis before and after granuloma evidence.

## Author contributions

DV and FJ conceptualized the review. DV prepared the first draft of the manuscript with MB (for imaging), J-FB (for pathology), EC (for microbiology), and BD (for drug-induced granulomatosis). MB provided the radiological figures and legends. J-FB provided the histological table and figures and legends. DV, MB, J-FB, EC, BD, CR, IB, AM, HN, J-MN, and FJ critically revised and edited the manuscript and agreed to the submitted version. All authors contributed to the article and approved the submitted version.

## Conflict of interest

The authors declare that this review was conducted in the absence of any commercial or financial relationships that could be construed as a potential conflict of interest.

## Publisher’s note

All claims expressed in this article are solely those of the authors and do not necessarily represent those of their affiliated organizations, or those of the publisher, the editors and the reviewers. Any product that may be evaluated in this article, or claim that may be made by its manufacturer, is not guaranteed or endorsed by the publisher.
